# Long-term safety and tolerability of donepezil 23 mg in patients with moderate to severe Alzheimer’s disease

**DOI:** 10.1186/1756-0500-5-283

**Published:** 2012-06-08

**Authors:** Pierre Tariot, Steven Salloway, Jane Yardley, Joan Mackell, Margaret Moline

**Affiliations:** 1 Banner Alzheimer’s Institute, 901 E. Willetta Street, Third Floor, Phoenix, AZ, 85006, USA; 2 Brown Alpert Medical School, 345 Blackstone Boulevard, Providence, RI, 02906, USA; 3 Neuroscience Product Creation Unit, Eisai Ltd, European Knowledge Centre, Mosquito Way, Hatfield, Hertfordshire, AL10 9SN, UK; 4 Pfizer, Inc, 235 E 42nd St 235/9/11, New York, NY, 10017, USA; 5 Eisai Medical Research, Eisai Inc, 55 Challenger Road, Ridgefield Park, NJ, 07660, USA

**Keywords:** Donepezil 23 mg, Donepezil, Safety, Tolerability, Clinical trial

## Abstract

**Background:**

Donepezil (23 mg/day) is approved by the US Food and Drug Administration for the treatment of patients with moderate to severe Alzheimer’s disease (AD). Approval was based on results from a 24-week, randomized, double-blind study of patients who were stable on donepezil 10 mg/day and randomized 2:1 to either increase their donepezil dose to 23 mg/day or continue taking 10 mg/day. The objective of this study was to assess the long-term safety and tolerability of donepezil 23 mg/day in patients with moderate to severe AD.

**Methods:**

Patients who completed the double-blind study and were eligible could enroll into a 12-month extension study of open-label donepezil 23 mg/day. Clinic visits took place at open-label baseline and at months 3, 6, 9, and 12. Safety analyses comprised examination of the incidence, severity, and timing of treatment-emergent adverse events (AEs); changes in weight, electrocardiogram, vital signs, and laboratory parameters; and discontinuation due to AEs.

**Results:**

915 double-blind study completers were enrolled in the open-label extension study and 902 comprised the safety population. Mean treatment duration in this study was 10.3 ± 3.5 months. In total, 674 patients (74.7%) reported at least one AE; in 320 of these patients (47.5%) at least one AE was considered to be possibly or probably study drug related. The majority of patients reporting AEs (81.9%) had AEs of mild or moderate severity. There were 268 patients (29.7%) who discontinued early, of which 123 (13.6%) were due to AEs.

Patients increasing donepezil dose from 10 mg/day in the double-blind study to 23 mg/day in the extension study had slightly higher rates of AEs and SAEs than patients who were already receiving 23 mg (78.0% and 16.9% vs 72.8% and 14.0%, respectively). The incidence of new AEs declined rapidly after the first 2 weeks and remained low throughout the duration of the study.

**Conclusion:**

This study shows that long-term treatment with donepezil 23 mg/day is associated with no new safety signals. The elevated incidence of AEs in patients increasing the dose of donepezil from 10 mg/day to 23 mg/day was limited to the initial weeks of the study.

## Background

Alzheimer’s disease (AD) is characterized by progressive impairment of cognitive and functional capacities [1,2]. Current treatments, which provide symptomatic benefits, include acetylcholinesterase inhibitors such as donepezil hydrochloride [3,4], and memantine, a glutamate receptor antagonist [5]. Despite optimal use of these therapies, symptomatic improvement is limited in magnitude and duration, after which patients continue to decline [6,7].

To address this clear medical need for more effective symptomatic therapy, a higher dose formulation of donepezil HCl (23 mg/day) was developed. The 23-mg dose is formulated as a matrix-type tablet, which provides a more gradual systemic absorption and longer time to maximum concentration compared with immediate release formulations [8]. The US FDA recently granted approval of donepezil 23 mg/day on the basis of findings from a 24-week, double-blind, randomized controlled trial in 1467 patients with moderate to severe AD [9] that demonstrated cognitive benefit when the dose of donepezil was increased to 23 mg/day compared with continuing on donepezil 10 mg/day.

Here, we report the results of the 1-year open-label extension of the double-blind trial, which evaluated the safety and tolerability of donepezil 23 mg/day in AD patients during long-term treatment.

## Methods

The study was conducted between 2007 and 2010 and included 191 investigators at 179 sites in Asia, Europe, North America, Oceania, South Africa, and South America.

### Patient population

The patient population in the double-blind study has been previously described [9]. Briefly, the study included males and females aged 45–90 years with a diagnosis of probable dementia of the Alzheimer’s type based on the National Institute of Neurological and Communicative Diseases and Stroke – Alzheimer’s Disease and Related Disorders criteria [10] and as defined in the Diagnostic and Statistical Manual of Mental Disorders Fourth Edition (code 290.00 or 290.10) [2]; a Mini-Mental State Evaluation (MMSE) score of 0–20 (moderate to severe impairment); a severe impairment battery (SIB) score [11] of ≤ 90 at baseline and screening; a Cornell Scale for Depression in Dementia [12] score < 12; and who were otherwise physically healthy. Patients had previously received donepezil 10 mg/day once daily for ≥ 12 weeks. Memantine ≤ 20 mg/day was permitted if the dose had been stable for ≥ 3 months. Patients were randomly assigned in a 2:1 ratio to receive either a high-dose donepezil tablet (23 mg once daily) or to remain on donepezil 10 mg/day.

Patients completing the double-blind study were eligible to enter the 12-month extension study if they had a competent caregiver, provided informed consent, and were without the following exclusion criteria: ongoing serious AEs (SAEs); a history of SAEs related to the study drug during the double-blind study; and no more than 3 days elapsed since completing the double-blind study.

### Study design

This was a 12-month open-label extension (ClinicalTrials.gov trial number NCT00566501) of the 24-week, randomized, double-blind, parallel-group study [9]. During the extension, all patients received donepezil 23 mg/day irrespective of the dose received during the double-blind phase. Clinic visits took place at baseline and at 3, 6, 9, and 12 months.

The protocol, all applicable amendments, and the informed consent form were approved by an Independent Ethics Committee (IEC) or Institutional Review Board (IRB). All participants provided informed consent before entering the open-label extension study. The trial was conducted in accordance with the principles of the Declaration of Helsinki and complied with local laws and regulations.

### Safety assessments

Safety was assessed at the final visit of the double-blind study (baseline visit of the extension study) and the 3-, 6-, 9-, and 12-month visits of the extension study. Treatment-emergent AEs (defined as events occurring after first dose of study drug in the extension study, or which began during the double-blind study and increased in severity during the extension study), serious AEs, and concomitant medication were recorded throughout the study. The investigator assessed AEs for severity (mild, moderate, severe) and possible or probable attribution to study drug. Vital signs, weight, and standard 12-lead electrocardiograms (ECG) were recorded, blood and urine samples were taken for clinical laboratory tests, and physical and neurological examinations were performed at each visit.

### Statistics and data analysis

Safety was analyzed for the safety population, which consisted of all patients who received at least one dose of donepezil 23 mg/day during the extension study and had at least one post-baseline safety assessment.

In addition, two patient subgroups were defined for the extension study according to whether patients had received donepezil 10 mg/day or donepezil 23 mg/day during the double-blind phase. These groups were designated the 10–23 subgroup and the 23–23 subgroup, respectively.

All AEs were coded using the Medical Dictionary for Regulatory Activities (MedDRA), Version 11.1. The numbers (percentages) of patients with at least one AE, at least one SAE, and at least one AE leading to discontinuation were summarized. AEs were summarized by preferred term, severity, and relationship to treatment; SAEs were summarized by preferred term and relationship to treatment. Deaths were evaluated for possible relationship to study drug. Duration of exposure to study drug was calculated for the extension study and for the double-blind and extension studies combined.

All safety-related observations were summarized using descriptive statistics. No inferential testing was performed.

## Results

### Demographics and baseline characteristics

Basic demographic characteristics are shown in Table 1 (Additional file 1). The mean age of patients was 74.3 ± 8.6 years. Just over one-third of the population was taking concomitant memantine at the start of the extension study.

**Table 1:** Baseline^†^* demographic characteristics

**Characteristic**	**Total**
Number of safety patients	902
Mean age (SD), years	74.3 (8.6)
Females, %	63.7
Race, %	
White	74.7
Asian/Pacific	15.5
Hispanic	6.7
Black	2.5
Other	0.6
Weight (kg), %	
< 55	22.9
55 to < 65	25.6
65 to < 75	25.5
≥ 75	25.9
Type of residence, %	
Lives alone	3.7
Lives with caregiver	80.2
Lives with relative or friend	9.4
Lives in retirement home or assisted living	4.5
Lives in intermediate or skilled nursing facility	1.4
Other	0.8
MMSE, mean (SD)	13.8 (5.9)
Memantine use, %	36.1

MMSE, Mini-Mental State Examination.

^†^*Extension study baseline.

### Patient disposition

**Figure 1:**
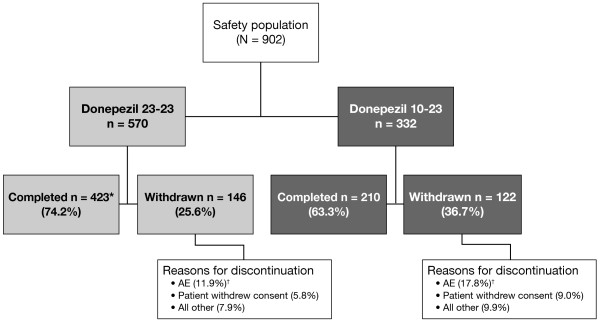
**Subject disposition.** *The completion status for one patient was not determined. ^†^Includes all treatment emergent and treatment non-emergent AEs and SAEs.

The patient disposition is shown in Figure 1 (Additional file 2).

Overall, 915 of the 1084 double-blind study completers were enrolled (84.4%) and 902 comprised the safety population. The reasons for non-enrollment were not analyzed. Within the safety population, 570 (63.2%) patients were in the 23–23 subgroup and 332 (36.8%) were in the 10–23 subgroup.

Mean duration of treatment in this study was 10.3 ± 3.5 months. Mean duration of treatment in the combined double-blind and extension studies was 15.9 ± 3.4 months. Mean study drug compliance rate for the safety population was 96.1%. Overall, there were 268 discontinuations (29.7%), of which 123 (13.6%) were due to AEs (Additional file 3).

### Safety and tolerability

In total, 674 patients (74.7%) reported at least one AE; in 320 of these patients (47.5%) at least one AE was considered to be possibly or probably study drug related. The majority of patients with one or more AE (552 of 674; 81.9%) reported AEs of mild or moderate severity. The AEs with the highest incidence were weight decreased, fall, agitation, urinary tract infection, and aggression (Table 2) (Additional file 4).

**Table 2:** Most frequently occurring adverse events (AE) in the safety population (incidence ≥ 3.0%)

**Preferred term**	**Percent of patients**
	**N = 902**
Patients with at least 1 AE	74.7
Weight decreased	11.2
Fall	8.8
Agitation	6.8
Urinary tract infection	5.9
Aggression	5.8
Diarrhea	4.3
Nausea	3.5
Hypertension	3.1
Syncope	3.1
Depression	3.0
Weight increased	3.0

The incidences of patients who reported AEs that are known to be associated with acetylcholinesterase inhibitors were: decreased weight (11.2%), weight loss ≥ 7% (11.1%), diarrhea (4.3%), nausea (3.5%), syncope (3.1%), vomiting (2.7%), anorexia (2.4%), bradycardia (1.2%), and gastrointestinal (GI) bleeding (0.8%, including the following terms: GI hemorrhage, hematemesis, hematochezia, melena, rectal bleeding, and upper GI hemorrhage). Of the 7 cases of GI bleeding, 5 occurred in subjects continuing on 23 mg from Study 326 (5/569, 0.9%) and 2 in those transitioning to 23 mg from 10 mg (2/330, 0.6%).

The patients in the 10–23 subgroup had higher rates of cholinergic AEs (nausea, vomiting, diarrhea) during the first 4 weeks of the study than those in the 23–23 subgroup (Table 3). Discontinuations due to AEs during this time period totaled 4.8% in the 10–23 subgroup, compared with 2.3% in the 23–23 subgroup. However, after this initial increase in AEs and discontinuations associated with the upward dose titration in the 10–23 subgroup, the incidence of patients with newly occurring AEs and discontinuations due to AEs declined rapidly and remained similarly low in both groups throughout the remainder of the study (Table 3).

**Table 3:** Cumulative incidence of adverse events (AEs) in the safety population by study week

	**Donepezil 23–23**				**Donepezil 10–23**			
	**N = 570**				**N = 332**			
	**1 week**	**2 weeks**	**4 weeks**	**52 weeks (total duration)**	**1 week**	**2 weeks**	**4 weeks**	**52 weeks (total duration)**
Patients with at least 1 AE, %	15.8	18.6	23.2	72.8	20.8	25.9	31.9	78.0
Patients who discontinued due to AEs, %	0.5	1.6	2.3	11.4	2.4	3.9	4.8	17.5
Patients with AEs, %								
Diarrhea	0.4	0.4	0.9	3.5	1.5	2.1	2.7	5.7
Nausea	0.2	0.2	0.2	2.1	3.9	4.2	4.2	6.0
Vomiting	0.0	0.0	0.0	1.6	2.4	2.4	2.7	4.5
Dizziness	0.4	0.4	0.5	1.1	0.9	1.2	1.5	3.6

SAEs were reported in 136 patients (15.1%) (Table 4) (Additional file 5). The most frequent SAEs were syncope, urinary tract infection, and fall. Of the SAEs occurring in > 1.0% of patients, only syncope occurred in a higher percentage of patients in the 10–23 subgroup (1.8%) than in the 23–23 subgroup (1.1%). Overall, 48 (5.3%) patients discontinued due to SAEs. The incidence of death in the safety population was 2.1% (n = 19); however, only one death, attributed to hematemesis, was assessed as probably related to study drug. Over the course of the study there were no changes in laboratory test values, vital signs, physical exam findings, neurologic findings, or ECG recordings indicative of a safety signal.

**Table 4:** Serious adverse events (SAEs) in the safety population (incidence ≥ 1.0%)

**Preferred term**	**Percent of patients N = 902**
Patients with at least 1 SAE	15.1
Occurring in ≥ 1% of patients	
Syncope	1.3
Urinary tract infection	1.2
Fall	1.0

## Discussion

Results from this 1-year open-label extension study show that donepezil 23 mg/day was generally well tolerated. No safety signals that had not been observed with donepezil 23 mg in the double-blind study were observed in this extension study, and no worsening of tolerability was observed during treatment with 23 mg for up to a further 12 months among patients who had already taken donepezil 23 mg for the prior 6 months. AEs that occurred most frequently were those that may be associated with AD itself, such as weight loss, falls, or agitation, and AEs typically associated with administration of cholinesterase inhibitors, such as gastrointestinal symptoms. Over 80% of all AEs were mild to moderate in severity and the rate of SAEs was similar to that observed in the double-blind study.

Weight loss was the most commonly observed AE over the course of the extension trial. In the absence of a control group, it is not possible to attribute this to study drug based on the findings from this study. Anorexia and weight loss are important considerations in AD patients, given their association with mortality [13]. The effect of donepezil on the body weight of AD patients has been studied in numerous trials and has been found to be similar to that observed in placebo-treated patients [14] (a total of 7–9% of donepezil- and 6–8% of placebo-treated patients experienced clinically significant weight loss [≥ 7% from baseline] in representative studies) [15-17]. The larger percentage noted in this study is likely due to the longer period of observation compared with most other studies, though it may also be related to the higher dose of donepezil and the severity of illness in this patient population. Weight loss is a concern in this population and should be monitored closely [13].

Bradycardia and GI bleeding are potentially serious AEs that have been associated with cholinesterase inhibitors. The rates of these AEs were very low in the extension study (1.2% and 0.8%, respectively). All cases of bradycardia resolved; one patient died of GI bleeding that was attributed to study drug. There was no notable difference in incidence of GI bleeding between the group continuing on 23 mg from the double-blind study and the group initiating 23 mg in this open-label study (0.9% and 0.6%, respectively), nor was the incidence different than that observed in the 23 mg group in the double-blind study (12/963 [1.1%]) [9]. Moreover, the fatality due to GI bleeding occurred in a patient who initiated 23 mg in this open-label study. Thus, it appears that the risk of GI bleeding in patients receiving donepezil 23 mg does not increase in incidence or severity over a longer duration of treatment with 23 mg.

Overall mortality in this 1-year study was 2.1%, consistent with that observed in the 6-month double-blind study (0.9%) and with the expected mortality in this population of elderly patients with moderate or severe AD [18].

Over the course of the study, 13.6% of patients discontinued due to AEs, including 17.5% of patients in the 10–23 subgroup and 11.4% of the patients in the 23–23 subgroup. This difference in rates of discontinuation appears to be attributed primarily to the transient increase in cholinergic-related gastrointestinal AEs (nausea, vomiting, diarrhea, and anorexia) in the 10–23 subgroup due to titration to the higher dose. This pattern was documented in the initial double-blind study, where 18.8% and 7.9% of patients discontinued in the donepezil 23 mg/day and 10 mg/day subgroups, respectively [9,19], and is consistent with other studies with a dose increase from donepezil 5 mg/day to 10 mg/day [8]. An increase in cholinergic AEs has been widely observed with acetylcholinesterase inhibitors when patients are titrated from a lower to a higher dose regimen [20,21]. After the initial peak, the likelihood of experiencing a GI-related AE for the first time during the study was low and similar in both subgroups. Strategies to manage AEs when increasing donepezil dose to 23 mg/day should be familiar to healthcare providers already treating patients with standard donepezil dosing regimens, and implementation of such strategies may reduce the incidence of early discontinuation.

## Conclusions

This 1-year extension study of donepezil 23 mg/day in patients with moderate or severe AD demonstrated no new safety findings and provides evidence for the acceptable long-term tolerability of this dosage option.

## Abbreviations

AD: Alzheimer’s disease; AE: Adverse event; MMSE: Mini-Mental State Evaluation; SIB: Severe impairment battery; SAE: Serious adverse event.

## Competing interests

Pierre Tariot has received consulting fees from Acadia, AC Immune, Allergan, Baxter Healthcare Corp., Eisai, Inc., Epix Pharmaceuticals, Forest Laboratories, Medavante, Novartis, Sanofi-Aventis, Schering-Plough, and Worldwide Clinical Trials; consulting fees and research support from Abbott Laboratories, AstraZeneca, Avid, Bristol Myers Squibb, Elan, Genentech, GlaxoSmithKline, Eli Lilly, Medivation, Merck, Pfizer Inc., Toyama, and Wyeth Laboratories; research support only from Baxter Healthcare Corp., and GE, and other research support from NIA, NIMH, Alzheimer’s Association, Arizona Department of Health Services, and the Institute for Mental Health Research. He has stock options in Medavante and Adamas and is listed as a contributor to a patent, “Biomarkers of Alzheimer’s Disease”.

Steven Salloway has provided consulting services to Eisai, Pfizer, Forest, Medivation, Myriad, Elan, Sanofi-Aventis, and Merck. He has received honoraria from Pfizer, Eisai, Novartis, Forest, and Elan. He has received research support from Eisai, Pfizer, Forest, Janssen, Myriad, Elan, Neurochem, Wyeth, Cephalon, the National Institutes of Health, and the Norman and Rosalie Fain Family Foundation.

Jane Yardley is an employee of Eisai Ltd.

Joan Mackell is an employee of Pfizer Inc.

Margaret Moline is a former employee of Eisai Inc.

## Authors’ contributions

PT participated in interpreting the study and helped draft the manuscript. JY analyzed and interpreted the data and helped draft the manuscript. MM conceived and designed the study, analyzed and interpreted the data, and helped draft the manuscript. JM participated in interpreting the study and helped draft the manuscript. SS participated in conducting and interpreting the study, and helped draft the manuscript. All authors read and approved the final manuscript.

## Supplementary Material

Additional file 1 Analysis Populations (Enrolled Subjects). Click here for file

Additional file 2 Summary of Treatment-Emergent Signs or Symptoms by Body System and Preferred Term (Safety Population). Click here for file

Additional file 3 Summary of Serious Treatment-Emergent Signs or Symptoms by Body System and Preferred Term (Safety Population). Click here for file

Additional file 4 Subject Baseline Demographic Characteristics (Safety Population). Click here for file

Additional file 5 Summary of Treatment Duration (Safety Population). Click here for file
