# Supplementary material for: Long-term safety and tolerability of donepezil 23 mg in patients with moderate to severe Alzheimer’s disease
Source: BMC Res Notes. 2012 Jun 8;5:283. doi: 10.1186/1756-0500-5-283 (PMC3493328; doi:10.1186/1756-0500-5-283)
Supplement: Additional file 1 — Analysis Populations (Enrolled Subjects). [file 1756-0500-5-283-S1.pdf]

Table 14.1.1.1
Analysis Populations
Enrolled Subjects

	Lead-in Treatment Group		
	Donepezil SR 23 mg<br>n (%)	Donepezil IR 10 mg<br>n (%)	Total<br>n (%)
Number of Subjects Enrolled	579	336	915
Safety Population	570 (100.0)	332 (100.0)	902 (100.0)
Intent-to-Treat Population	560 (98.2)	321 (96.7)	881 (97.7)

Data Source: Listing 16.2.3.1
Note: Number of subjects in the Safety Population is used as the denominator for computing percentages.

tad_ap.sas 25MAY2010 22:15

Eisai Inc.
Protocol: E2020-G000-328
Investigator: Multicenter

Table 14.1.1.2.1
Subject Disposition
Enrolled Subjects

	Lead-in Treatment Group		
	Donepezil SR 23 mg<br>n (%)	Donepezil IR 10 mg<br>n (%)	Total<br>n (%)
Number of Subjects Enrolled	579	336	915
Safety Population	570	332	902
Completed	423 (74.2)	210 (63.3)	633 (70.2)
Discontinued	146 (25.6)	122 (36.7)	268 (29.7)
Not Determined [1]	1 (0.2)	0 (0.0)	1 (0.1)
Reason for Discontinuation			
Adverse Event [2]	68 (11.9)	59 (17.8)	127 (14.1)
Intercurrent Illness	0 (0.0)	0 (0.0)	0 (0.0)
Medication Non-compliance	5 (0.9)	0 (0.0)	5 (0.6)
Protocol Violation	2 (0.4)	2 (0.6)	4 (0.4)
Request of Investigator or Sponsor	6 (1.1)	7 (2.1)	13 (1.4)
Patient Withdrew Consent	33 (5.8)	30 (9.0)	63 (7.0)
Lack of Efficacy	8 (1.4)	3 (0.9)	11 (1.2)
Other	24 (4.2)	21 (6.3)	45 (5.0)
Deaths	12 (2.1)	5 (1.5)	17 (1.9)

Data Source: Listing 14.3.2.1.1, Listing 16.2.1.1, Listing 16.2.1.2
Note: Number of subjects in the Safety Population is used as the denominator for computing percentages.
[1] Subject is considered Not Determined when the final termination data is not available.
[2] Includes serious adverse events.

tad_ds1.sas 25MAY2010 22:16

Table 14.1.1.2.2
Subject Disposition
ITT Population

	Lead-in Treatment Group		
	Donepezil SR 23 mg<br>n (%)	Donepezil IR 10 mg<br>n (%)	Total<br>n (%)
ITT Population	560	321	881
Completed	423 (75.5)	210 (65.4)	633 (71.9)
Discontinued	136 (24.3)	111 (34.6)	247 (28.0)
Not Determined [1]	1 (0.2)	0 (0.0)	1 (0.1)
Reason for Discontinuation			
Adverse Event [2]	62 (11.1)	51 (15.9)	113 (12.8)
Intercurrent Illness	0 (0.0)	0 (0.0)	0 (0.0)
Medication Non-compliance	4 (0.7)	0 (0.0)	4 (0.5)
Protocol Violation	2 (0.4)	1 (0.3)	3 (0.3)
Request of Investigator or Sponsor	6 (1.1)	7 (2.2)	13 (1.5)
Patient Withdrew Consent	33 (5.9)	29 (9.0)	62 (7.0)
Lack of Efficacy	8 (1.4)	3 (0.9)	11 (1.2)
Other	21 (3.8)	20 (6.2)	41 (4.7)
Deaths	10 (1.8)	3 (0.9)	13 (1.5)

Data Source: Listing 14.3.2.1.1, Listing 16.2.1.1, Listing 16.2.1.2
Note: Number of subjects in the ITT Population is used as the denominator for computing percentages.
[1] Subject is considered Not Determined when the final termination data is not available.
[2] Includes serious adverse events.

tad_ds2.sas 25MAY2010 22:16
