# Supplementary material for: Long-term safety and tolerability of donepezil 23 mg in patients with moderate to severe Alzheimer’s disease
Source: BMC Res Notes. 2012 Jun 8;5:283. doi: 10.1186/1756-0500-5-283 (PMC3493328; doi:10.1186/1756-0500-5-283)
Supplement: Additional file 2 — Summary of Treatment-Emergent Signs or Symptoms by Body System and Preferred Term (Safety Population). [file 1756-0500-5-283-S2.pdf]

Table 14.3.1.4
Summary of Treatment-Emergent Signs or Symptoms by Body System and Preferred Term
Safety Population

	Lead-in Treatment Group		
Body System<br>Preferred Term	Donepezil SR 23 mg<br>n (%)	Donepezil IR 10 mg<br>n (%)	Total<br>n (%)
Number of Subjects	570	332	902
Number of Subjects with at Least One Treatment-Emergent Sign or Symptom	415 (72.8)	259 (78.0)	674 (74.7)
Blood and lymphatic system disorders	8 (1.4)	16 (4.8)	24 (2.7)
Anaemia	3 (0.5)	9 (2.7)	12 (1.3)
Anisocytosis	0 (0.0)	1 (0.3)	1 (0.1)
Hypercoagulation	1 (0.2)	0 (0.0)	1 (0.1)
Hypochromasia	0 (0.0)	1 (0.3)	1 (0.1)
Iron deficiency anaemia	0 (0.0)	3 (0.9)	3 (0.3)
Leukocytosis	1 (0.2)	0 (0.0)	1 (0.1)
Leukopenia	1 (0.2)	1 (0.3)	2 (0.2)
Lymphadenopathy	1 (0.2)	2 (0.6)	3 (0.3)
Neutropenia	1 (0.2)	0 (0.0)	1 (0.1)
Poikilocytosis	0 (0.0)	1 (0.3)	1 (0.1)
Polychromasia	0 (0.0)	1 (0.3)	1 (0.1)
Thrombocytopenia	0 (0.0)	2 (0.6)	2 (0.2)

Data Source: Listing 16.2.3.1, Listing 16.2.7.1
A treatment-emergent sign or symptom (TESS) is defined as an adverse event that either (1) begins on or after the date of the first dose of study drug of E2020-G000-328 [up to 30 days after date of last dose of study drug of E2020-G000-328] or (2) increases in severity during the treatment period.
Subjects are counted only once per treatment in each row.
Number of subjects in Safety Population is used as the denominator for computing percentages.

Table 14.3.1.4
Summary of Treatment-Emergent Signs or Symptoms by Body System and Preferred Term
Safety Population

Body System<br>Preferred Term	Lead-in Treatment Group<br>Donepezil SR 23 mg<br>n (%)	 Donepezil IR 10 mg<br>n (%)	Total<br>n (%)
Number of Subjects	570	332	902
Number of Subjects with at Least One Treatment-Emergent Sign or Symptom	415 (72.8)	259 (78.0)	674 (74.7)
Cardiac disorders	30 (5.3)	23 (6.9)	53 (5.9)
Acute myocardial infarction	0 (0.0)	1 (0.3)	1 (0.1)
Angina pectoris	3 (0.5)	1 (0.3)	4 (0.4)
Arrhythmia supraventricular	0 (0.0)	1 (0.3)	1 (0.1)
Atrial fibrillation	2 (0.4)	2 (0.6)	4 (0.4)
Atrial flutter	1 (0.2)	0 (0.0)	1 (0.1)
Atrioventricular block complete	1 (0.2)	0 (0.0)	1 (0.1)
Atrioventricular block first degree	2 (0.4)	2 (0.6)	4 (0.4)
Bradycardia	5 (0.9)	6 (1.8)	11 (1.2)
Bundle branch block left	0 (0.0)	1 (0.3)	1 (0.1)
Bundle branch block right	1 (0.2)	0 (0.0)	1 (0.1)
Cardiac failure	2 (0.4)	1 (0.3)	3 (0.3)
Cardio-respiratory arrest	1 (0.2)	0 (0.0)	1 (0.1)
Coronary artery disease	1 (0.2)	1 (0.3)	2 (0.2)

Data Source: Listing 16.2.3.1, Listing 16.2.7.1
A treatment-emergent sign or symptom (TESS) is defined as an adverse event that either (1) begins on or after the date of the first dose of study drug of E2020-G000-328 [up to 30 days after date of last dose of study drug of E2020-G000-328] or (2) increases in severity during the treatment period.
Subjects are counted only once per treatment in each row.
Number of subjects in Safety Population is used as the denominator for computing percentages.

Table 14.3.1.4
Summary of Treatment-Emergent Signs or Symptoms by Body System and Preferred Term
Safety Population

Body System<br>Preferred Term	Lead-in Treatment Group<br>Donepezil SR 23 mg<br>n (%)	 Donepezil IR 10 mg<br>n (%)	Total<br>n (%)
Number of Subjects	570	332	902
Number of Subjects with at Least One Treatment-Emergent Sign or Symptom	415 (72.8)	259 (78.0)	674 (74.7)
Cardiac disorders (Continued)			
Myocardial infarction	3 (0.5)	0 (0.0)	3 (0.3)
Myocardial ischaemia	4 (0.7)	1 (0.3)	5 (0.6)
Sinus bradycardia	4 (0.7)	2 (0.6)	6 (0.7)
Sinus tachycardia	1 (0.2)	2 (0.6)	3 (0.3)
Supraventricular extrasystoles	0 (0.0)	3 (0.9)	3 (0.3)
Tachycardia	0 (0.0)	1 (0.3)	1 (0.1)
Ventricular extrasystoles	1 (0.2)	1 (0.3)	2 (0.2)
Ventricular tachycardia	1 (0.2)	0 (0.0)	1 (0.1)
Congenital, familial and genetic disorders	1 (0.2)	1 (0.3)	2 (0.2)
Encephalocele	0 (0.0)	1 (0.3)	1 (0.1)
Hydrocele	1 (0.2)	0 (0.0)	1 (0.1)
Meningocele	0 (0.0)	1 (0.3)	1 (0.1)

Data Source: Listing 16.2.3.1, Listing 16.2.7.1
A treatment-emergent sign or symptom (TESS) is defined as an adverse event that either (1) begins on or after the date of the first dose of study drug of E2020-G000-328 [up to 30 days after date of last dose of study drug of E2020-G000-328] or (2) increases in severity during the treatment period.
Subjects are counted only once per treatment in each row.
Number of subjects in Safety Population is used as the denominator for computing percentages.

Table 14.3.1.4
Summary of Treatment-Emergent Signs or Symptoms by Body System and Preferred Term
Safety Population

	Lead-in Treatment Group		
Body System<br>Preferred Term	Donepezil SR 23 mg<br>n (%)	Donepezil IR 10 mg<br>n (%)	Total<br>n (%)
Number of Subjects	570	332	902
Number of Subjects with at Least One Treatment-Emergent Sign or Symptom	415 (72.8)	259 (78.0)	674 (74.7)
Ear and labyrinth disorders	11 (1.9)	4 (1.2)	15 (1.7)
Cerumen impaction	1 (0.2)	0 (0.0)	1 (0.1)
Deafness	0 (0.0)	1 (0.3)	1 (0.1)
Hypoacusis	2 (0.4)	0 (0.0)	2 (0.2)
Motion sickness	0 (0.0)	1 (0.3)	1 (0.1)
Tinnitus	1 (0.2)	0 (0.0)	1 (0.1)
Vertigo	6 (1.1)	1 (0.3)	7 (0.8)
Vertigo positional	1 (0.2)	1 (0.3)	2 (0.2)
Endocrine disorders	5 (0.9)	1 (0.3)	6 (0.7)
Goitre	0 (0.0)	1 (0.3)	1 (0.1)
Hypoparathyroidism	1 (0.2)	0 (0.0)	1 (0.1)
Hypothyroidism	4 (0.7)	0 (0.0)	4 (0.4)

Data Source: Listing 16.2.3.1, Listing 16.2.7.1
A treatment-emergent sign or symptom (TESS) is defined as an adverse event that either (1) begins on or after the date of the first dose of study drug of E2020-G000-328 [up to 30 days after date of last dose of study drug of E2020-G000-328] or (2) increases in severity during the treatment period.
Subjects are counted only once per treatment in each row.
Number of subjects in Safety Population is used as the denominator for computing percentages.

Table 14.3.1.4
Summary of Treatment-Emergent Signs or Symptoms by Body System and Preferred Term
Safety Population

	Lead-in Treatment Group		
Body System<br>Preferred Term	Donepezil SR 23 mg<br>n (%)	Donepezil IR 10 mg<br>n (%)	Total<br>n (%)
Number of Subjects	570	332	902
Number of Subjects with at Least One Treatment-Emergent Sign or Symptom	415 (72.8)	259 (78.0)	674 (74.7)
Eye disorders	15 (2.6)	7 (2.1)	22 (2.4)
Blepharitis	1 (0.2)	0 (0.0)	1 (0.1)
Blepharospasm	1 (0.2)	0 (0.0)	1 (0.1)
Cataract	1 (0.2)	2 (0.6)	3 (0.3)
Conjunctival haemorrhage	0 (0.0)	1 (0.3)	1 (0.1)
Conjunctivitis	3 (0.5)	0 (0.0)	3 (0.3)
Conjunctivitis allergic	0 (0.0)	1 (0.3)	1 (0.1)
Dry eye	0 (0.0)	1 (0.3)	1 (0.1)
Entropion	0 (0.0)	1 (0.3)	1 (0.1)
Eye irritation	1 (0.2)	0 (0.0)	1 (0.1)
Eyelid ptosis	1 (0.2)	0 (0.0)	1 (0.1)
Glaucoma	2 (0.4)	0 (0.0)	2 (0.2)
Heterophoria	1 (0.2)	0 (0.0)	1 (0.1)
Lacrimal disorder	1 (0.2)	0 (0.0)	1 (0.1)

Data Source: Listing 16.2.3.1, Listing 16.2.7.1
A treatment-emergent sign or symptom (TESS) is defined as an adverse event that either (1) begins on or after the date of the first dose of study drug of E2020-G000-328 [up to 30 days after date of last dose of study drug of E2020-G000-328] or (2) increases in severity during the treatment period.
Subjects are counted only once per treatment in each row.
Number of subjects in Safety Population is used as the denominator for computing percentages.

tad_ae.sas 25MAY2010 22:15

Table 14.3.1.4
Summary of Treatment-Emergent Signs or Symptoms by Body System and Preferred Term
Safety Population

Body System<br>Preferred Term	Lead-in Treatment Group<br>Donepezil SR 23 mg<br>n (%)	 Donepezil IR 10 mg<br>n (%)	Total<br>n (%)
Number of Subjects	570	332	902
Number of Subjects with at Least One Treatment-Emergent Sign or Symptom	415 (72.8)	259 (78.0)	674 (74.7)
Eye disorders (Continued)			
Lacrimation increased	1 (0.2)	0 (0.0)	1 (0.1)
Macular degeneration	1 (0.2)	0 (0.0)	1 (0.1)
Miosis	1 (0.2)	0 (0.0)	1 (0.1)
Ocular hyperaemia	1 (0.2)	0 (0.0)	1 (0.1)
Visual acuity reduced	0 (0.0)	1 (0.3)	1 (0.1)
Gastrointestinal disorders	75 (13.2)	64 (19.3)	139 (15.4)
Abdominal discomfort	0 (0.0)	1 (0.3)	1 (0.1)
Abdominal distension	1 (0.2)	0 (0.0)	1 (0.1)
Abdominal hernia	1 (0.2)	1 (0.3)	2 (0.2)
Abdominal pain	6 (1.1)	7 (2.1)	13 (1.4)
Abdominal pain upper	1 (0.2)	2 (0.6)	3 (0.3)
Abdominal tenderness	1 (0.2)	1 (0.3)	2 (0.2)

Data Source: Listing 16.2.3.1, Listing 16.2.7.1
A treatment-emergent sign or symptom (TESS) is defined as an adverse event that either (1) begins on or after the date of the first dose of study drug of E2020-G000-328 [up to 30 days after date of last dose of study drug of E2020-G000-328] or (2) increases in severity during the treatment period.
Subjects are counted only once per treatment in each row.
Number of subjects in Safety Population is used as the denominator for computing percentages.

Table 14.3.1.4
Summary of Treatment-Emergent Signs or Symptoms by Body System and Preferred Term
Safety Population

Body System<br>Preferred Term	Lead-in Treatment Group<br>Donepezil SR 23 mg<br>n (%)	 Donepezil IR 10 mg<br>n (%)	Total<br>n (%)
Number of Subjects	570	332	902
Number of Subjects with at Least One Treatment-Emergent Sign or Symptom	415 (72.8)	259 (78.0)	674 (74.7)
Gastrointestinal disorders (Continued)			
Acquired oesophageal web	0 (0.0)	1 (0.3)	1 (0.1)
Anorectal discomfort	1 (0.2)	0 (0.0)	1 (0.1)
Breath odour	1 (0.2)	0 (0.0)	1 (0.1)
Colitis	1 (0.2)	0 (0.0)	1 (0.1)
Constipation	6 (1.1)	8 (2.4)	14 (1.6)
Defaecation urgency	0 (0.0)	1 (0.3)	1 (0.1)
Dental caries	0 (0.0)	1 (0.3)	1 (0.1)
Diarrhoea	20 (3.5)	19 (5.7)	39 (4.3)
Diverticulum	1 (0.2)	0 (0.0)	1 (0.1)
Diverticulum intestinal	0 (0.0)	1 (0.3)	1 (0.1)
Dry mouth	1 (0.2)	0 (0.0)	1 (0.1)
Duodenal ulcer perforation	1 (0.2)	0 (0.0)	1 (0.1)
Dyspepsia	1 (0.2)	2 (0.6)	3 (0.3)

Data Source: Listing 16.2.3.1, Listing 16.2.7.1
A treatment-emergent sign or symptom (TESS) is defined as an adverse event that either (1) begins on or after the date of the first dose of study drug of E2020-G000-328 [up to 30 days after date of last dose of study drug of E2020-G000-328] or (2) increases in severity during the treatment period.
Subjects are counted only once per treatment in each row.
Number of subjects in Safety Population is used as the denominator for computing percentages.

tad_ae.sas 25MAY2010 22:15

Table 14.3.1.4
Summary of Treatment-Emergent Signs or Symptoms by Body System and Preferred Term
Safety Population

	Lead-in Treatment Group		
Body System<br>Preferred Term	Donepezil SR 23 mg<br>n (%)	Donepezil IR 10 mg<br>n (%)	Total<br>n (%)
Number of Subjects	570	332	902
Number of Subjects with at Least One Treatment-Emergent Sign or Symptom	415 (72.8)	259 (78.0)	674 (74.7)
Gastrointestinal disorders (Continued)			
Dysphagia	3 (0.5)	2 (0.6)	5 (0.6)
Eructation	0 (0.0)	1 (0.3)	1 (0.1)
Faecal incontinence	7 (1.2)	4 (1.2)	11 (1.2)
Faecaloma	0 (0.0)	1 (0.3)	1 (0.1)
Flatulence	0 (0.0)	1 (0.3)	1 (0.1)
Frequent bowel movements	0 (0.0)	1 (0.3)	1 (0.1)
Gastric ulcer	0 (0.0)	1 (0.3)	1 (0.1)
Gastritis	3 (0.5)	1 (0.3)	4 (0.4)
Gastrointestinal haemorrhage	1 (0.2)	0 (0.0)	1 (0.1)
Gastrooesophageal reflux disease	4 (0.7)	2 (0.6)	6 (0.7)
Haematemesis	0 (0.0)	2 (0.6)	2 (0.2)
Haematochezia	1 (0.2)	0 (0.0)	1 (0.1)
Haemorrhoidal haemorrhage	1 (0.2)	0 (0.0)	1 (0.1)

Data Source: Listing 16.2.3.1, Listing 16.2.7.1
A treatment-emergent sign or symptom (TESS) is defined as an adverse event that either (1) begins on or after the date of the first dose of study drug of E2020-G000-328 [up to 30 days after date of last dose of study drug of E2020-G000-328] or (2) increases in severity during the treatment period.
Subjects are counted only once per treatment in each row.
Number of subjects in Safety Population is used as the denominator for computing percentages.

tad_ae.sas 25MAY2010 22:15

Table 14.3.1.4
Summary of Treatment-Emergent Signs or Symptoms by Body System and Preferred Term
Safety Population

Body System<br>Preferred Term	Lead-in Treatment Group<br>Donepezil SR 23 mg<br>n (%)	 Donepezil IR 10 mg<br>n (%)	Total<br>n (%)
Number of Subjects	570	332	902
Number of Subjects with at Least One Treatment-Emergent Sign or Symptom	415 (72.8)	259 (78.0)	674 (74.7)
Gastrointestinal disorders (Continued)			
Haemorrhoids	2 (0.4)	0 (0.0)	2 (0.2)
Hiatus hernia	3 (0.5)	1 (0.3)	4 (0.4)
Ileus paralytic	1 (0.2)	0 (0.0)	1 (0.1)
Melaena	1 (0.2)	0 (0.0)	1 (0.1)
Mouth ulceration	1 (0.2)	0 (0.0)	1 (0.1)
Nausea	12 (2.1)	20 (6.0)	32 (3.5)
Oesophageal ulcer	1 (0.2)	0 (0.0)	1 (0.1)
Pancreatic mass	1 (0.2)	0 (0.0)	1 (0.1)
Rectal haemorrhage	0 (0.0)	1 (0.3)	1 (0.1)
Rectal prolapse	1 (0.2)	0 (0.0)	1 (0.1)
Rectal tenesmus	0 (0.0)	1 (0.3)	1 (0.1)
Stomach discomfort	0 (0.0)	1 (0.3)	1 (0.1)
Subileus	1 (0.2)	0 (0.0)	1 (0.1)

Data Source: Listing 16.2.3.1, Listing 16.2.7.1
A treatment-emergent sign or symptom (TESS) is defined as an adverse event that either (1) begins on or after the date of the first dose of study drug of E2020-G000-328 [up to 30 days after date of last dose of study drug of E2020-G000-328] or (2) increases in severity during the treatment period.
Subjects are counted only once per treatment in each row.
Number of subjects in Safety Population is used as the denominator for computing percentages.

tad_ae.sas 25MAY2010 22:15

Table 14.3.1.4
Summary of Treatment-Emergent Signs or Symptoms by Body System and Preferred Term
Safety Population

Body System<br>Preferred Term	Lead-in Treatment Group<br>Donepezil SR 23 mg<br>n (%)	 Donepezil IR 10 mg<br>n (%)	Total<br>n (%)
Number of Subjects	570	332	902
Number of Subjects with at Least One Treatment-Emergent Sign or Symptom	415 (72.8)	259 (78.0)	674 (74.7)
Gastrointestinal disorders (Continued)			
Tongue coated	1 (0.2)	0 (0.0)	1 (0.1)
Tooth loss	1 (0.2)	0 (0.0)	1 (0.1)
Upper gastrointestinal haemorrhage	1 (0.2)	0 (0.0)	1 (0.1)
Vomiting	9 (1.6)	15 (4.5)	24 (2.7)
General disorders and administration site conditions	56 (9.8)	34 (10.2)	90 (10.0)
Asthenia	6 (1.1)	12 (3.6)	18 (2.0)
Chest pain	1 (0.2)	0 (0.0)	1 (0.1)
Death	2 (0.4)	0 (0.0)	2 (0.2)
Fatigue	5 (0.9)	3 (0.9)	8 (0.9)
Feeling cold	0 (0.0)	1 (0.3)	1 (0.1)
Gait disturbance	7 (1.2)	4 (1.2)	11 (1.2)
Irritability	13 (2.3)	7 (2.1)	20 (2.2)

Data Source: Listing 16.2.3.1, Listing 16.2.7.1
A treatment-emergent sign or symptom (TESS) is defined as an adverse event that either (1) begins on or after the date of the first dose of study drug of E2020-G000-328 [up to 30 days after date of last dose of study drug of E2020-G000-328] or (2) increases in severity during the treatment period.
Subjects are counted only once per treatment in each row.
Number of subjects in Safety Population is used as the denominator for computing percentages.

tad_ae.sas 25MAY2010 22:15

Table 14.3.1.4
Summary of Treatment-Emergent Signs or Symptoms by Body System and Preferred Term
Safety Population

	Lead-in Treatment Group		
Body System<br>Preferred Term	Donepezil SR 23 mg<br>n (%)	Donepezil IR 10 mg<br>n (%)	Total<br>n (%)
Number of Subjects	570	332	902
Number of Subjects with at Least One Treatment-Emergent Sign or Symptom	415 (72.8)	259 (78.0)	674 (74.7)
General disorders and administration site conditions (Continued)			
Local swelling	1 (0.2)	0 (0.0)	1 (0.1)
Malaise	1 (0.2)	1 (0.3)	2 (0.2)
Multi-organ failure	0 (0.0)	1 (0.3)	1 (0.1)
Non-cardiac chest pain	3 (0.5)	0 (0.0)	3 (0.3)
Oedema peripheral	15 (2.6)	5 (1.5)	20 (2.2)
Pitting oedema	1 (0.2)	0 (0.0)	1 (0.1)
Pyrexia	7 (1.2)	2 (0.6)	9 (1.0)
Sudden death	0 (0.0)	1 (0.3)	1 (0.1)
Tenderness	1 (0.2)	0 (0.0)	1 (0.1)
Vessel puncture site haematoma	0 (0.0)	1 (0.3)	1 (0.1)
Hepatobiliary disorders	1 (0.2)	3 (0.9)	4 (0.4)
Cholecystitis acute	0 (0.0)	1 (0.3)	1 (0.1)

Data Source: Listing 16.2.3.1, Listing 16.2.7.1
A treatment-emergent sign or symptom (TESS) is defined as an adverse event that either (1) begins on or after the date of the first dose of study drug of E2020-G000-328 [up to 30 days after date of last dose of study drug of E2020-G000-328] or (2) increases in severity during the treatment period.
Subjects are counted only once per treatment in each row.
Number of subjects in Safety Population is used as the denominator for computing percentages.

tad_ae.sas 25MAY2010 22:15

Table 14.3.1.4
Summary of Treatment-Emergent Signs or Symptoms by Body System and Preferred Term
Safety Population

Body System<br>Preferred Term	Lead-in Treatment Group<br>Donepezil SR 23 mg<br>n (%)	 Donepezil IR 10 mg<br>n (%)	Total<br>n (%)
Number of Subjects	570	332	902
Number of Subjects with at Least One Treatment-Emergent Sign or Symptom	415 (72.8)	259 (78.0)	674 (74.7)
Hepatobiliary disorders (Continued)			
Cholelithiasis	1 (0.2)	1 (0.3)	2 (0.2)
Hepatic cirrhosis	0 (0.0)	1 (0.3)	1 (0.1)
Immune system disorders	1 (0.2)	1 (0.3)	2 (0.2)
Hypersensitivity	1 (0.2)	0 (0.0)	1 (0.1)
Multiple allergies	0 (0.0)	1 (0.3)	1 (0.1)
Infections and infestations	107 (18.8)	63 (19.0)	170 (18.8)
Abdominal abscess	1 (0.2)	0 (0.0)	1 (0.1)
Abscess jaw	1 (0.2)	0 (0.0)	1 (0.1)
Acarodermatitis	1 (0.2)	0 (0.0)	1 (0.1)
Bacteriuria	1 (0.2)	0 (0.0)	1 (0.1)
Body tinea	1 (0.2)	0 (0.0)	1 (0.1)

Data Source: Listing 16.2.3.1, Listing 16.2.7.1
A treatment-emergent sign or symptom (TESS) is defined as an adverse event that either (1) begins on or after the date of the first dose of study drug of E2020-G000-328 [up to 30 days after date of last dose of study drug of E2020-G000-328] or (2) increases in severity during the treatment period.
Subjects are counted only once per treatment in each row.
Number of subjects in Safety Population is used as the denominator for computing percentages.

tad_ae.sas 25MAY2010 22:15

Table 14.3.1.4
Summary of Treatment-Emergent Signs or Symptoms by Body System and Preferred Term
Safety Population

	Lead-in Treatment Group		
Body System<br>Preferred Term	Donepezil SR 23 mg<br>n (%)	Donepezil IR 10 mg<br>n (%)	Total<br>n (%)
Number of Subjects	570	332	902
Number of Subjects with at Least One Treatment-Emergent Sign or Symptom	415 (72.8)	259 (78.0)	674 (74.7)
Infections and infestations (Continued)			
Bronchitis	11 (1.9)	4 (1.2)	15 (1.7)
Cellulitis	3 (0.5)	0 (0.0)	3 (0.3)
Cellulitis staphylococcal	1 (0.2)	0 (0.0)	1 (0.1)
Cystitis	1 (0.2)	4 (1.2)	5 (0.6)
Ear infection	2 (0.4)	0 (0.0)	2 (0.2)
Eye infection	1 (0.2)	0 (0.0)	1 (0.1)
Fungal skin infection	1 (0.2)	2 (0.6)	3 (0.3)
Gastroenteritis	5 (0.9)	3 (0.9)	8 (0.9)
Gastroenteritis viral	1 (0.2)	0 (0.0)	1 (0.1)
Gastrointestinal infection	0 (0.0)	1 (0.3)	1 (0.1)
Herpes zoster	3 (0.5)	0 (0.0)	3 (0.3)
Hordeolum	0 (0.0)	1 (0.3)	1 (0.1)
Infected skin ulcer	1 (0.2)	0 (0.0)	1 (0.1)

Data Source: Listing 16.2.3.1, Listing 16.2.7.1
A treatment-emergent sign or symptom (TESS) is defined as an adverse event that either (1) begins on or after the date of the first dose of study drug of E2020-G000-328 [up to 30 days after date of last dose of study drug of E2020-G000-328] or (2) increases in severity during the treatment period.
Subjects are counted only once per treatment in each row.
Number of subjects in Safety Population is used as the denominator for computing percentages.

tad_ae.sas 25MAY2010 22:15

Table 14.3.1.4
Summary of Treatment-Emergent Signs or Symptoms by Body System and Preferred Term
Safety Population

	Lead-in Treatment Group		
Body System<br>Preferred Term	Donepezil SR 23 mg<br>n (%)	Donepezil IR 10 mg<br>n (%)	Total<br>n (%)
Number of Subjects	570	332	902
Number of Subjects with at Least One Treatment-Emergent Sign or Symptom	415 (72.8)	259 (78.0)	674 (74.7)
Infections and infestations (Continued)			
Influenza	4 (0.7)	4 (1.2)	8 (0.9)
Liver abscess	0 (0.0)	1 (0.3)	1 (0.1)
Lobar pneumonia	0 (0.0)	1 (0.3)	1 (0.1)
Localised infection	1 (0.2)	1 (0.3)	2 (0.2)
Lower respiratory tract infection	3 (0.5)	3 (0.9)	6 (0.7)
Lung infection	0 (0.0)	1 (0.3)	1 (0.1)
Nasopharyngitis	14 (2.5)	11 (3.3)	25 (2.8)
Onychomycosis	1 (0.2)	0 (0.0)	1 (0.1)
Oral infection	1 (0.2)	0 (0.0)	1 (0.1)
Orchitis	1 (0.2)	0 (0.0)	1 (0.1)
Otitis externa	1 (0.2)	0 (0.0)	1 (0.1)
Paronychia	1 (0.2)	0 (0.0)	1 (0.1)
Peritonsillar abscess	1 (0.2)	0 (0.0)	1 (0.1)

Data Source: Listing 16.2.3.1, Listing 16.2.7.1
A treatment-emergent sign or symptom (TESS) is defined as an adverse event that either (1) begins on or after the date of the first dose of study drug of E2020-G000-328 [up to 30 days after date of last dose of study drug of E2020-G000-328] or (2) increases in severity during the treatment period.
Subjects are counted only once per treatment in each row.
Number of subjects in Safety Population is used as the denominator for computing percentages.

tad_ae.sas 25MAY2010 22:15

Table 14.3.1.4
Summary of Treatment-Emergent Signs or Symptoms by Body System and Preferred Term
Safety Population

Body System<br>Preferred Term	Lead-in Treatment Group<br>Donepezil SR 23 mg<br>n (%)	 Donepezil IR 10 mg<br>n (%)	Total<br>n (%)
Number of Subjects	570	332	902
Number of Subjects with at Least One Treatment-Emergent Sign or Symptom	415 (72.8)	259 (78.0)	674 (74.7)
Infections and infestations (Continued)			
Pharyngitis	1 (0.2)	0 (0.0)	1 (0.1)
Pharyngitis bacterial	1 (0.2)	0 (0.0)	1 (0.1)
Pneumonia	7 (1.2)	3 (0.9)	10 (1.1)
Rash pustular	2 (0.4)	0 (0.0)	2 (0.2)
Respiratory tract infection	0 (0.0)	1 (0.3)	1 (0.1)
Rhinitis	1 (0.2)	2 (0.6)	3 (0.3)
Sepsis	2 (0.4)	0 (0.0)	2 (0.2)
Sinusitis	1 (0.2)	0 (0.0)	1 (0.1)
Skin infection	1 (0.2)	0 (0.0)	1 (0.1)
Tracheobronchitis	1 (0.2)	0 (0.0)	1 (0.1)
Tuberculosis	1 (0.2)	0 (0.0)	1 (0.1)
Upper respiratory tract infection	7 (1.2)	6 (1.8)	13 (1.4)
Urethritis	1 (0.2)	0 (0.0)	1 (0.1)

Data Source: Listing 16.2.3.1, Listing 16.2.7.1
A treatment-emergent sign or symptom (TESS) is defined as an adverse event that either (1) begins on or after the date of the first dose of study drug of E2020-G000-328 [up to 30 days after date of last dose of study drug of E2020-G000-328] or (2) increases in severity during the treatment period.
Subjects are counted only once per treatment in each row.
Number of subjects in Safety Population is used as the denominator for computing percentages.

tad_ae.sas 25MAY2010 22:15

Table 14.3.1.4
Summary of Treatment-Emergent Signs or Symptoms by Body System and Preferred Term
Safety Population

Body System<br>Preferred Term	Lead-in Treatment Group<br>Donepezil SR 23 mg<br>n (%)	 Donepezil IR 10 mg<br>n (%)	Total<br>n (%)
Number of Subjects	570	332	902
Number of Subjects with at Least One Treatment-Emergent Sign or Symptom	415 (72.8)	259 (78.0)	674 (74.7)
Infections and infestations (Continued)			
Urinary tract infection	33 (5.8)	20 (6.0)	53 (5.9)
Viral infection	0 (0.0)	1 (0.3)	1 (0.1)
Viral upper respiratory tract infection	1 (0.2)	1 (0.3)	2 (0.2)
Vulvovaginal mycotic infection	1 (0.2)	0 (0.0)	1 (0.1)
Vulvovaginitis	0 (0.0)	1 (0.3)	1 (0.1)
Injury, poisoning and procedural complications	82 (14.4)	38 (11.4)	120 (13.3)
Accidental overdose	7 (1.2)	3 (0.9)	10 (1.1)
Acetabulum fracture	0 (0.0)	1 (0.3)	1 (0.1)
Animal bite	1 (0.2)	0 (0.0)	1 (0.1)
Back injury	1 (0.2)	0 (0.0)	1 (0.1)
Collapse of lung	0 (0.0)	1 (0.3)	1 (0.1)
Compression fracture	1 (0.2)	0 (0.0)	1 (0.1)

Data Source: Listing 16.2.3.1, Listing 16.2.7.1
A treatment-emergent sign or symptom (TESS) is defined as an adverse event that either (1) begins on or after the date of the first dose of study drug of E2020-G000-328 [up to 30 days after date of last dose of study drug of E2020-G000-328] or (2) increases in severity during the treatment period.
Subjects are counted only once per treatment in each row.
Number of subjects in Safety Population is used as the denominator for computing percentages.

tad_ae.sas 25MAY2010 22:15

Table 14.3.1.4
Summary of Treatment-Emergent Signs or Symptoms by Body System and Preferred Term
Safety Population

	Lead-in Treatment Group		
Body System<br>Preferred Term	Donepezil SR 23 mg<br>n (%)	Donepezil IR 10 mg<br>n (%)	Total<br>n (%)
Number of Subjects	570	332	902
Number of Subjects with at Least One Treatment-Emergent Sign or Symptom	415 (72.8)	259 (78.0)	674 (74.7)
Injury, poisoning and procedural complications (Continued)			
Concussion	1 (0.2)	0 (0.0)	1 (0.1)
Contusion	9 (1.6)	7 (2.1)	16 (1.8)
Device lead damage	1 (0.2)	0 (0.0)	1 (0.1)
Dislocation of joint prosthesis	0 (0.0)	1 (0.3)	1 (0.1)
Excoriation	2 (0.4)	1 (0.3)	3 (0.3)
Eye injury	1 (0.2)	0 (0.0)	1 (0.1)
Face injury	1 (0.2)	0 (0.0)	1 (0.1)
Facial bones fracture	3 (0.5)	1 (0.3)	4 (0.4)
Fall	54 (9.5)	25 (7.5)	79 (8.8)
Femoral neck fracture	3 (0.5)	0 (0.0)	3 (0.3)
Femur fracture	2 (0.4)	0 (0.0)	2 (0.2)
Hand fracture	2 (0.4)	0 (0.0)	2 (0.2)
Head injury	6 (1.1)	1 (0.3)	7 (0.8)

Data Source: Listing 16.2.3.1, Listing 16.2.7.1
A treatment-emergent sign or symptom (TESS) is defined as an adverse event that either (1) begins on or after the date of the first dose of study drug of E2020-G000-328 [up to 30 days after date of last dose of study drug of E2020-G000-328] or (2) increases in severity during the treatment period.
Subjects are counted only once per treatment in each row.
Number of subjects in Safety Population is used as the denominator for computing percentages.

tad_ae.sas 25MAY2010 22:15

Table 14.3.1.4
Summary of Treatment-Emergent Signs or Symptoms by Body System and Preferred Term
Safety Population

Body System<br>Preferred Term	Lead-in Treatment Group<br>Donepezil SR 23 mg<br>n (%)	 Donepezil IR 10 mg<br>n (%)	Total<br>n (%)
Number of Subjects	570	332	902
Number of Subjects with at Least One Treatment-Emergent Sign or Symptom	415 (72.8)	259 (78.0)	674 (74.7)
Injury, poisoning and procedural complications (Continued)			
Hip fracture	2 (0.4)	1 (0.3)	3 (0.3)
Humerus fracture	1 (0.2)	0 (0.0)	1 (0.1)
Jaw fracture	0 (0.0)	1 (0.3)	1 (0.1)
Joint dislocation	1 (0.2)	0 (0.0)	1 (0.1)
Joint injury	1 (0.2)	0 (0.0)	1 (0.1)
Joint sprain	3 (0.5)	0 (0.0)	3 (0.3)
Laceration	2 (0.4)	3 (0.9)	5 (0.6)
Lumbar vertebral fracture	1 (0.2)	0 (0.0)	1 (0.1)
Mouth injury	1 (0.2)	0 (0.0)	1 (0.1)
Muscle strain	1 (0.2)	1 (0.3)	2 (0.2)
Overdose	4 (0.7)	4 (1.2)	8 (0.9)
Periorbital haematoma	1 (0.2)	1 (0.3)	2 (0.2)
Procedural pain	1 (0.2)	0 (0.0)	1 (0.1)

Data Source: Listing 16.2.3.1, Listing 16.2.7.1
A treatment-emergent sign or symptom (TESS) is defined as an adverse event that either (1) begins on or after the date of the first dose of study drug of E2020-G000-328 [up to 30 days after date of last dose of study drug of E2020-G000-328] or (2) increases in severity during the treatment period.
Subjects are counted only once per treatment in each row.
Number of subjects in Safety Population is used as the denominator for computing percentages.

tad_ae.sas 25MAY2010 22:15

Table 14.3.1.4
Summary of Treatment-Emergent Signs or Symptoms by Body System and Preferred Term
Safety Population

Body System<br>Preferred Term	Lead-in Treatment Group<br>Donepezil SR 23 mg<br>n (%)	 Donepezil IR 10 mg<br>n (%)	Total<br>n (%)
Number of Subjects	570	332	902
Number of Subjects with at Least One Treatment-Emergent Sign or Symptom	415 (72.8)	259 (78.0)	674 (74.7)
Injury, poisoning and procedural complications (Continued)			
Radius fracture	0 (0.0)	1 (0.3)	1 (0.1)
Rib fracture	1 (0.2)	0 (0.0)	1 (0.1)
Road traffic accident	1 (0.2)	0 (0.0)	1 (0.1)
Scratch	1 (0.2)	0 (0.0)	1 (0.1)
Skin laceration	10 (1.8)	6 (1.8)	16 (1.8)
Spinal fracture	1 (0.2)	0 (0.0)	1 (0.1)
Subdural haematoma	1 (0.2)	3 (0.9)	4 (0.4)
Subdural haemorrhage	1 (0.2)	0 (0.0)	1 (0.1)
Thoracic vertebral fracture	1 (0.2)	0 (0.0)	1 (0.1)
Tooth fracture	0 (0.0)	1 (0.3)	1 (0.1)
Traumatic haematoma	1 (0.2)	0 (0.0)	1 (0.1)
Upper limb fracture	1 (0.2)	1 (0.3)	2 (0.2)
Vertebral injury	0 (0.0)	1 (0.3)	1 (0.1)

Data Source: Listing 16.2.3.1, Listing 16.2.7.1
A treatment-emergent sign or symptom (TESS) is defined as an adverse event that either (1) begins on or after the date of the first dose of study drug of E2020-G000-328 [up to 30 days after date of last dose of study drug of E2020-G000-328] or (2) increases in severity during the treatment period.
Subjects are counted only once per treatment in each row.
Number of subjects in Safety Population is used as the denominator for computing percentages.

tad_ae.sas 25MAY2010 22:15

Table 14.3.1.4
Summary of Treatment-Emergent Signs or Symptoms by Body System and Preferred Term
Safety Population

Body System<br>Preferred Term	Lead-in Treatment Group<br>Donepezil SR 23 mg<br>n (%)	 Donepezil IR 10 mg<br>n (%)	Total<br>n (%)
Number of Subjects	570	332	902
Number of Subjects with at Least One Treatment-Emergent Sign or Symptom	415 (72.8)	259 (78.0)	674 (74.7)
Injury, poisoning and procedural complications (Continued)			
Wound	0 (0.0)	1 (0.3)	1 (0.1)
Wrist fracture	1 (0.2)	0 (0.0)	1 (0.1)
Investigations	107 (18.8)	73 (22.0)	180 (20.0)
Alanine aminotransferase increased	2 (0.4)	1 (0.3)	3 (0.3)
Aspartate aminotransferase increased	2 (0.4)	1 (0.3)	3 (0.3)
Bacteria urine identified	0 (0.0)	1 (0.3)	1 (0.1)
Blood alkaline phosphatase increased	2 (0.4)	2 (0.6)	4 (0.4)
Blood cholesterol increased	3 (0.5)	2 (0.6)	5 (0.6)
Blood creatine phosphokinase MB increased	1 (0.2)	1 (0.3)	2 (0.2)
Blood creatine phosphokinase increased	8 (1.4)	5 (1.5)	13 (1.4)
Blood creatinine increased	2 (0.4)	0 (0.0)	2 (0.2)
Blood folate decreased	0 (0.0)	1 (0.3)	1 (0.1)

Data Source: Listing 16.2.3.1, Listing 16.2.7.1
A treatment-emergent sign or symptom (TESS) is defined as an adverse event that either (1) begins on or after the date of the first dose of study drug of E2020-G000-328 [up to 30 days after date of last dose of study drug of E2020-G000-328] or (2) increases in severity during the treatment period.
Subjects are counted only once per treatment in each row.
Number of subjects in Safety Population is used as the denominator for computing percentages.

tad_ae.sas 25MAY2010 22:15

Table 14.3.1.4
Summary of Treatment-Emergent Signs or Symptoms by Body System and Preferred Term
Safety Population

	Lead-in Treatment Group		
Body System<br>Preferred Term	Donepezil SR 23 mg<br>n (%)	Donepezil IR 10 mg<br>n (%)	Total<br>n (%)
Number of Subjects	570	332	902
Number of Subjects with at Least One Treatment-Emergent Sign or Symptom	415 (72.8)	259 (78.0)	674 (74.7)
Investigations (Continued)			
Blood glucose increased	1 (0.2)	1 (0.3)	2 (0.2)
Blood lactate dehydrogenase increased	0 (0.0)	1 (0.3)	1 (0.1)
Blood pressure diastolic increased	1 (0.2)	0 (0.0)	1 (0.1)
Blood pressure increased	0 (0.0)	2 (0.6)	2 (0.2)
Blood triglycerides increased	4 (0.7)	0 (0.0)	4 (0.4)
Blood urea increased	3 (0.5)	1 (0.3)	4 (0.4)
Blood urine present	2 (0.4)	0 (0.0)	2 (0.2)
Crystal urine present	1 (0.2)	0 (0.0)	1 (0.1)
Electrocardiogram QT prolonged	3 (0.5)	1 (0.3)	4 (0.4)
Electrocardiogram ST-T change	1 (0.2)	0 (0.0)	1 (0.1)
Electrocardiogram ST-T segment abnormal	1 (0.2)	0 (0.0)	1 (0.1)
Electrocardiogram T wave abnormal	0 (0.0)	1 (0.3)	1 (0.1)
Electrocardiogram T wave inversion	0 (0.0)	1 (0.3)	1 (0.1)

Data Source: Listing 16.2.3.1, Listing 16.2.7.1
A treatment-emergent sign or symptom (TESS) is defined as an adverse event that either (1) begins on or after the date of the first dose of study drug of E2020-G000-328 [up to 30 days after date of last dose of study drug of E2020-G000-328] or (2) increases in severity during the treatment period.
Subjects are counted only once per treatment in each row.
Number of subjects in Safety Population is used as the denominator for computing percentages.

tad_ae.sas 25MAY2010 22:15

Table 14.3.1.4
Summary of Treatment-Emergent Signs or Symptoms by Body System and Preferred Term
Safety Population

Body System<br>Preferred Term	Lead-in Treatment Group<br>Donepezil SR 23 mg<br>n (%)	 Donepezil IR 10 mg<br>n (%)	Total<br>n (%)
Number of Subjects	570	332	902
Number of Subjects with at Least One Treatment-Emergent Sign or Symptom	415 (72.8)	259 (78.0)	674 (74.7)
Investigations (Continued)			
Electrocardiogram repolarisation abnormality	1 (0.2)	0 (0.0)	1 (0.1)
Eosinophil count increased	0 (0.0)	1 (0.3)	1 (0.1)
Haematocrit decreased	2 (0.4)	1 (0.3)	3 (0.3)
Haemoglobin decreased	1 (0.2)	1 (0.3)	2 (0.2)
Hepatic enzyme increased	2 (0.4)	0 (0.0)	2 (0.2)
Lipase increased	1 (0.2)	1 (0.3)	2 (0.2)
Low density lipoprotein increased	1 (0.2)	0 (0.0)	1 (0.1)
Monocyte count decreased	1 (0.2)	0 (0.0)	1 (0.1)
Neutrophil count increased	0 (0.0)	1 (0.3)	1 (0.1)
Neutrophil hypersegmented morphology present	0 (0.0)	1 (0.3)	1 (0.1)
Neutrophil percentage increased	1 (0.2)	0 (0.0)	1 (0.1)
Neutrophil toxic granulation present	0 (0.0)	1 (0.3)	1 (0.1)
Pedal pulse decreased	1 (0.2)	1 (0.3)	2 (0.2)

Data Source: Listing 16.2.3.1, Listing 16.2.7.1
A treatment-emergent sign or symptom (TESS) is defined as an adverse event that either (1) begins on or after the date of the first dose of study drug of E2020-G000-328 [up to 30 days after date of last dose of study drug of E2020-G000-328] or (2) increases in severity during the treatment period.
Subjects are counted only once per treatment in each row.
Number of subjects in Safety Population is used as the denominator for computing percentages.

tad_ae.sas 25MAY2010 22:15

Table 14.3.1.4
Summary of Treatment-Emergent Signs or Symptoms by Body System and Preferred Term
Safety Population

Body System<br>Preferred Term	Lead-in Treatment Group<br>Donepezil SR 23 mg<br>n (%)	 Donepezil IR 10 mg<br>n (%)	Total<br>n (%)
Number of Subjects	570	332	902
Number of Subjects with at Least One Treatment-Emergent Sign or Symptom	415 (72.8)	259 (78.0)	674 (74.7)
Investigations (Continued)			
Platelet count increased	0 (0.0)	1 (0.3)	1 (0.1)
Precancerous cells present	1 (0.2)	0 (0.0)	1 (0.1)
Prostatic specific antigen increased	0 (0.0)	1 (0.3)	1 (0.1)
Protein urine present	1 (0.2)	0 (0.0)	1 (0.1)
Pulse abnormal	1 (0.2)	0 (0.0)	1 (0.1)
Pulse absent	0 (0.0)	1 (0.3)	1 (0.1)
Red blood cell count decreased	1 (0.2)	1 (0.3)	2 (0.2)
Red blood cell elliptocytes present	0 (0.0)	1 (0.3)	1 (0.1)
Transaminases increased	1 (0.2)	0 (0.0)	1 (0.1)
Tuberculin test positive	1 (0.2)	0 (0.0)	1 (0.1)
Urine analysis abnormal	1 (0.2)	0 (0.0)	1 (0.1)
Vitamin B12 decreased	0 (0.0)	2 (0.6)	2 (0.2)
Weight decreased	58 (10.2)	43 (13.0)	101 (11.2)
Weight increased	15 (2.6)	12 (3.6)	27 (3.0)

Data Source: Listing 16.2.3.1, Listing 16.2.7.1
A treatment-emergent sign or symptom (TESS) is defined as an adverse event that either (1) begins on or after the date of the first dose of study drug of E2020-G000-328 [up to 30 days after date of last dose of study drug of E2020-G000-328] or (2) increases in severity during the treatment period.
Subjects are counted only once per treatment in each row.
Number of subjects in Safety Population is used as the denominator for computing percentages.

tad_ae.sas 25MAY2010 22:15

Table 14.3.1.4
Summary of Treatment-Emergent Signs or Symptoms by Body System and Preferred Term
Safety Population

Body System<br>Preferred Term	Lead-in Treatment Group<br>Donepezil SR 23 mg<br>n (%)	 Donepezil IR 10 mg<br>n (%)	Total<br>n (%)
Number of Subjects	570	332	902
Number of Subjects with at Least One Treatment-Emergent Sign or Symptom	415 (72.8)	259 (78.0)	674 (74.7)
Metabolism and nutrition disorders	57 (10.0)	47 (14.2)	104 (11.5)
Anorexia	11 (1.9)	11 (3.3)	22 (2.4)
Decreased appetite	7 (1.2)	4 (1.2)	11 (1.2)
Dehydration	8 (1.4)	5 (1.5)	13 (1.4)
Diabetes mellitus	6 (1.1)	2 (0.6)	8 (0.9)
Diabetes mellitus inadequate control	1 (0.2)	1 (0.3)	2 (0.2)
Dyslipidaemia	1 (0.2)	0 (0.0)	1 (0.1)
Glucose tolerance impaired	1 (0.2)	0 (0.0)	1 (0.1)
Gout	0 (0.0)	2 (0.6)	2 (0.2)
Hypercalcaemia	1 (0.2)	1 (0.3)	2 (0.2)
Hypercholesterolaemia	10 (1.8)	7 (2.1)	17 (1.9)
Hyperglycaemia	6 (1.1)	3 (0.9)	9 (1.0)
Hyperlipidaemia	1 (0.2)	4 (1.2)	5 (0.6)
Hypernatraemia	1 (0.2)	1 (0.3)	2 (0.2)

Data Source: Listing 16.2.3.1, Listing 16.2.7.1
A treatment-emergent sign or symptom (TESS) is defined as an adverse event that either (1) begins on or after the date of the first dose of study drug of E2020-G000-328 [up to 30 days after date of last dose of study drug of E2020-G000-328] or (2) increases in severity during the treatment period.
Subjects are counted only once per treatment in each row.
Number of subjects in Safety Population is used as the denominator for computing percentages.

tad_ae.sas 25MAY2010 22:15

Table 14.3.1.4
Summary of Treatment-Emergent Signs or Symptoms by Body System and Preferred Term
Safety Population

Body System<br>Preferred Term	Lead-in Treatment Group<br>Donepezil SR 23 mg<br>n (%)	 Donepezil IR 10 mg<br>n (%)	Total<br>n (%)
Number of Subjects	570	332	902
Number of Subjects with at Least One Treatment-Emergent Sign or Symptom	415 (72.8)	259 (78.0)	674 (74.7)
Metabolism and nutrition disorders (Continued)			
Hyperphagia	1 (0.2)	0 (0.0)	1 (0.1)
Hypertriglyceridaemia	3 (0.5)	1 (0.3)	4 (0.4)
Hypocalcaemia	0 (0.0)	1 (0.3)	1 (0.1)
Hypoglycaemia	1 (0.2)	1 (0.3)	2 (0.2)
Hypokalaemia	3 (0.5)	2 (0.6)	5 (0.6)
Hyponatraemia	2 (0.4)	4 (1.2)	6 (0.7)
Hypophagia	0 (0.0)	2 (0.6)	2 (0.2)
Increased appetite	0 (0.0)	2 (0.6)	2 (0.2)
Obesity	1 (0.2)	0 (0.0)	1 (0.1)
Overweight	1 (0.2)	0 (0.0)	1 (0.1)
Type 2 diabetes mellitus	2 (0.4)	2 (0.6)	4 (0.4)
Vitamin D deficiency	2 (0.4)	1 (0.3)	3 (0.3)
Vitamin E deficiency	1 (0.2)	0 (0.0)	1 (0.1)

Data Source: Listing 16.2.3.1, Listing 16.2.7.1
A treatment-emergent sign or symptom (TESS) is defined as an adverse event that either (1) begins on or after the date of the first dose of study drug of E2020-G000-328 [up to 30 days after date of last dose of study drug of E2020-G000-328] or (2) increases in severity during the treatment period.
Subjects are counted only once per treatment in each row.
Number of subjects in Safety Population is used as the denominator for computing percentages.

tad_ae.sas 25MAY2010 22:15

Table 14.3.1.4
Summary of Treatment-Emergent Signs or Symptoms by Body System and Preferred Term
Safety Population

	Lead-in Treatment Group		
Body System<br>Preferred Term	Donepezil SR 23 mg<br>n (%)	Donepezil IR 10 mg<br>n (%)	Total<br>n (%)
Number of Subjects	570	332	902
Number of Subjects with at Least One Treatment-Emergent Sign or Symptom	415 (72.8)	259 (78.0)	674 (74.7)
Musculoskeletal and connective tissue disorders	38 (6.7)	35 (10.5)	73 (8.1)
Arthralgia	7 (1.2)	8 (2.4)	15 (1.7)
Arthritis	1 (0.2)	2 (0.6)	3 (0.3)
Back pain	8 (1.4)	5 (1.5)	13 (1.4)
Intervertebral disc protrusion	0 (0.0)	1 (0.3)	1 (0.1)
Joint stiffness	1 (0.2)	0 (0.0)	1 (0.1)
Joint swelling	0 (0.0)	1 (0.3)	1 (0.1)
Kyphosis	0 (0.0)	1 (0.3)	1 (0.1)
Muscle atrophy	1 (0.2)	0 (0.0)	1 (0.1)
Muscle rigidity	2 (0.4)	5 (1.5)	7 (0.8)
Muscle spasms	1 (0.2)	3 (0.9)	4 (0.4)
Muscle twitching	1 (0.2)	0 (0.0)	1 (0.1)
Musculoskeletal chest pain	2 (0.4)	0 (0.0)	2 (0.2)
Musculoskeletal pain	3 (0.5)	2 (0.6)	5 (0.6)

Data Source: Listing 16.2.3.1, Listing 16.2.7.1
A treatment-emergent sign or symptom (TESS) is defined as an adverse event that either (1) begins on or after the date of the first dose of study drug of E2020-G000-328 [up to 30 days after date of last dose of study drug of E2020-G000-328] or (2) increases in severity during the treatment period.
Subjects are counted only once per treatment in each row.
Number of subjects in Safety Population is used as the denominator for computing percentages.

tad_ae.sas 25MAY2010 22:15

Table 14.3.1.4
Summary of Treatment-Emergent Signs or Symptoms by Body System and Preferred Term
Safety Population

	Lead-in Treatment Group		
Body System<br>Preferred Term	Donepezil SR 23 mg<br>n (%)	Donepezil IR 10 mg<br>n (%)	Total<br>n (%)
Number of Subjects	570	332	902
Number of Subjects with at Least One Treatment-Emergent Sign or Symptom	415 (72.8)	259 (78.0)	674 (74.7)
Musculoskeletal and connective tissue disorders (Continued)			
Musculoskeletal stiffness	2 (0.4)	0 (0.0)	2 (0.2)
Myalgia	0 (0.0)	1 (0.3)	1 (0.1)
Neck mass	1 (0.2)	0 (0.0)	1 (0.1)
Neck pain	0 (0.0)	2 (0.6)	2 (0.2)
Nuchal rigidity	0 (0.0)	1 (0.3)	1 (0.1)
Osteoarthritis	6 (1.1)	2 (0.6)	8 (0.9)
Osteoporosis	3 (0.5)	2 (0.6)	5 (0.6)
Pain in extremity	1 (0.2)	5 (1.5)	6 (0.7)
Pathological fracture	1 (0.2)	0 (0.0)	1 (0.1)
Periarthritis	1 (0.2)	0 (0.0)	1 (0.1)
Posture abnormal	1 (0.2)	1 (0.3)	2 (0.2)
Rotator cuff syndrome	1 (0.2)	0 (0.0)	1 (0.1)
Spinal column stenosis	1 (0.2)	0 (0.0)	1 (0.1)

Data Source: Listing 16.2.3.1, Listing 16.2.7.1
A treatment-emergent sign or symptom (TESS) is defined as an adverse event that either (1) begins on or after the date of the first dose of study drug of E2020-G000-328 [up to 30 days after date of last dose of study drug of E2020-G000-328] or (2) increases in severity during the treatment period.
Subjects are counted only once per treatment in each row.
Number of subjects in Safety Population is used as the denominator for computing percentages.

tad_ae.sas 25MAY2010 22:15

Table 14.3.1.4
Summary of Treatment-Emergent Signs or Symptoms by Body System and Preferred Term
Safety Population

	Lead-in Treatment Group		
Body System<br>Preferred Term	Donepezil SR 23 mg<br>n (%)	Donepezil IR 10 mg<br>n (%)	Total<br>n (%)
Number of Subjects	570	332	902
Number of Subjects with at Least One Treatment-Emergent Sign or Symptom	415 (72.8)	259 (78.0)	674 (74.7)
Musculoskeletal and connective tissue disorders (Continued)			
Spondylitis	1 (0.2)	1 (0.3)	2 (0.2)
Synovial cyst	1 (0.2)	0 (0.0)	1 (0.1)
Neoplasms benign, malignant and unspecified (incl cysts and polyps)	12 (2.1)	7 (2.1)	19 (2.1)
Basal cell carcinoma	2 (0.4)	1 (0.3)	3 (0.3)
Breast cancer	1 (0.2)	0 (0.0)	1 (0.1)
Colon adenoma	1 (0.2)	0 (0.0)	1 (0.1)
Haemangioma of liver	1 (0.2)	0 (0.0)	1 (0.1)
Lipoma	0 (0.0)	1 (0.3)	1 (0.1)
Lung carcinoma cell type unspecified stage IV	1 (0.2)	0 (0.0)	1 (0.1)
Lung neoplasm	1 (0.2)	0 (0.0)	1 (0.1)
Lung neoplasm malignant	1 (0.2)	1 (0.3)	2 (0.2)
Malignant melanoma	0 (0.0)	1 (0.3)	1 (0.1)

Data Source: Listing 16.2.3.1, Listing 16.2.7.1
A treatment-emergent sign or symptom (TESS) is defined as an adverse event that either (1) begins on or after the date of the first dose of study drug of E2020-G000-328 [up to 30 days after date of last dose of study drug of E2020-G000-328] or (2) increases in severity during the treatment period.
Subjects are counted only once per treatment in each row.
Number of subjects in Safety Population is used as the denominator for computing percentages.

tad_ae.sas 25MAY2010 22:15

Table 14.3.1.4
Summary of Treatment-Emergent Signs or Symptoms by Body System and Preferred Term
Safety Population

Body System<br>Preferred Term	Lead-in Treatment Group<br>Donepezil SR 23 mg<br>n (%)	 Donepezil IR 10 mg<br>n (%)	Total<br>n (%)
Number of Subjects	570	332	902
Number of Subjects with at Least One Treatment-Emergent Sign or Symptom	415 (72.8)	259 (78.0)	674 (74.7)
Neoplasms benign, malignant and unspecified (incl cysts and polyps) (Continued)			
Melanoma recurrent	0 (0.0)	1 (0.3)	1 (0.1)
Metastases to liver	1 (0.2)	0 (0.0)	1 (0.1)
Metastases to spine	0 (0.0)	1 (0.3)	1 (0.1)
Metastasis	1 (0.2)	0 (0.0)	1 (0.1)
Prostate cancer	1 (0.2)	2 (0.6)	3 (0.3)
Prostate cancer stage II	0 (0.0)	1 (0.3)	1 (0.1)
Rectal cancer	1 (0.2)	0 (0.0)	1 (0.1)
Renal neoplasm	1 (0.2)	0 (0.0)	1 (0.1)
Nervous system disorders	102 (17.9)	78 (23.5)	180 (20.0)
Ageusia	1 (0.2)	0 (0.0)	1 (0.1)
Agnosia	1 (0.2)	0 (0.0)	1 (0.1)
Akathisia	0 (0.0)	1 (0.3)	1 (0.1)

Data Source: Listing 16.2.3.1, Listing 16.2.7.1
A treatment-emergent sign or symptom (TESS) is defined as an adverse event that either (1) begins on or after the date of the first dose of study drug of E2020-G000-328 [up to 30 days after date of last dose of study drug of E2020-G000-328] or (2) increases in severity during the treatment period.
Subjects are counted only once per treatment in each row.
Number of subjects in Safety Population is used as the denominator for computing percentages.

tad_ae.sas 25MAY2010 22:15

Table 14.3.1.4
Summary of Treatment-Emergent Signs or Symptoms by Body System and Preferred Term
Safety Population

	Lead-in Treatment Group		
Body System<br>Preferred Term	Donepezil SR 23 mg<br>n (%)	Donepezil IR 10 mg<br>n (%)	Total<br>n (%)
Number of Subjects	570	332	902
Number of Subjects with at Least One Treatment-Emergent Sign or Symptom	415 (72.8)	259 (78.0)	674 (74.7)
Nervous system disorders (Continued)			
Akinesia	2 (0.4)	1 (0.3)	3 (0.3)
Altered state of consciousness	1 (0.2)	0 (0.0)	1 (0.1)
Aphasia	8 (1.4)	4 (1.2)	12 (1.3)
Apraxia	2 (0.4)	1 (0.3)	3 (0.3)
Ataxia	0 (0.0)	1 (0.3)	1 (0.1)
Balance disorder	2 (0.4)	0 (0.0)	2 (0.2)
Bradykinesia	1 (0.2)	1 (0.3)	2 (0.2)
Brain oedema	0 (0.0)	1 (0.3)	1 (0.1)
Cerebral atrophy	1 (0.2)	0 (0.0)	1 (0.1)
Cerebral haemorrhage	1 (0.2)	1 (0.3)	2 (0.2)
Cerebral infarction	0 (0.0)	1 (0.3)	1 (0.1)
Cerebrospinal fistula	0 (0.0)	1 (0.3)	1 (0.1)
Cerebrovascular accident	5 (0.9)	3 (0.9)	8 (0.9)

Data Source: Listing 16.2.3.1, Listing 16.2.7.1
A treatment-emergent sign or symptom (TESS) is defined as an adverse event that either (1) begins on or after the date of the first dose of study drug of E2020-G000-328 [up to 30 days after date of last dose of study drug of E2020-G000-328] or (2) increases in severity during the treatment period.
Subjects are counted only once per treatment in each row.
Number of subjects in Safety Population is used as the denominator for computing percentages.

Table 14.3.1.4
Summary of Treatment-Emergent Signs or Symptoms by Body System and Preferred Term
Safety Population

	Lead-in Treatment Group		
Body System<br>Preferred Term	Donepezil SR 23 mg<br>n (%)	Donepezil IR 10 mg<br>n (%)	Total<br>n (%)
Number of Subjects	570	332	902
Number of Subjects with at Least One Treatment-Emergent Sign or Symptom	415 (72.8)	259 (78.0)	674 (74.7)
Nervous system disorders (Continued)			
Cognitive disorder	5 (0.9)	3 (0.9)	8 (0.9)
Convulsion	2 (0.4)	0 (0.0)	2 (0.2)
Dementia	0 (0.0)	1 (0.3)	1 (0.1)
Dementia Alzheimer's type	3 (0.5)	4 (1.2)	7 (0.8)
Disturbance in attention	5 (0.9)	2 (0.6)	7 (0.8)
Dizziness	6 (1.1)	12 (3.6)	18 (2.0)
Drooling	1 (0.2)	0 (0.0)	1 (0.1)
Dysarthria	4 (0.7)	0 (0.0)	4 (0.4)
Dyskinesia	1 (0.2)	1 (0.3)	2 (0.2)
Encephalopathy	1 (0.2)	0 (0.0)	1 (0.1)
Epilepsy	1 (0.2)	1 (0.3)	2 (0.2)
Extrapyramidal disorder	1 (0.2)	2 (0.6)	3 (0.3)
Headache	6 (1.1)	5 (1.5)	11 (1.2)

Data Source: Listing 16.2.3.1, Listing 16.2.7.1
A treatment-emergent sign or symptom (TESS) is defined as an adverse event that either (1) begins on or after the date of the first dose of study drug of E2020-G000-328 [up to 30 days after date of last dose of study drug of E2020-G000-328] or (2) increases in severity during the treatment period.
Subjects are counted only once per treatment in each row.
Number of subjects in Safety Population is used as the denominator for computing percentages.

tad_ae.sas 25MAY2010 22:15

Table 14.3.1.4
Summary of Treatment-Emergent Signs or Symptoms by Body System and Preferred Term
Safety Population

Body System<br>Preferred Term	Lead-in Treatment Group<br>Donepezil SR 23 mg<br>n (%)	 Donepezil IR 10 mg<br>n (%)	Total<br>n (%)
Number of Subjects	570	332	902
Number of Subjects with at Least One Treatment-Emergent Sign or Symptom	415 (72.8)	259 (78.0)	674 (74.7)
Nervous system disorders (Continued)			
Hemiparesis	1 (0.2)	1 (0.3)	2 (0.2)
Hemiplegia	0 (0.0)	1 (0.3)	1 (0.1)
Hemisensory neglect	0 (0.0)	1 (0.3)	1 (0.1)
Hydrocephalus	1 (0.2)	0 (0.0)	1 (0.1)
Hypersomnia	0 (0.0)	1 (0.3)	1 (0.1)
Hypokinesia	1 (0.2)	0 (0.0)	1 (0.1)
Hypotonia	1 (0.2)	0 (0.0)	1 (0.1)
Intention tremor	1 (0.2)	0 (0.0)	1 (0.1)
Ischaemic cerebral infarction	0 (0.0)	1 (0.3)	1 (0.1)
Ischaemic stroke	0 (0.0)	1 (0.3)	1 (0.1)
Lacunar infarction	0 (0.0)	1 (0.3)	1 (0.1)
Lethargy	1 (0.2)	1 (0.3)	2 (0.2)
Loss of consciousness	1 (0.2)	1 (0.3)	2 (0.2)

Data Source: Listing 16.2.3.1, Listing 16.2.7.1
A treatment-emergent sign or symptom (TESS) is defined as an adverse event that either (1) begins on or after the date of the first dose of study drug of E2020-G000-328 [up to 30 days after date of last dose of study drug of E2020-G000-328] or (2) increases in severity during the treatment period.
Subjects are counted only once per treatment in each row.
Number of subjects in Safety Population is used as the denominator for computing percentages.

Table 14.3.1.4

Summary of Treatment-Emergent Signs or Symptoms by Body System and Preferred Term

Safety Population

Body System<br>Preferred Term	Lead-in Treatment Group<br>Donepezil SR 23 mg<br>n (%)	 Donepezil IR 10 mg<br>n (%)	Total<br>n (%)
Number of Subjects	570	332	902
Number of Subjects with at Least One Treatment-Emergent Sign or Symptom	415 (72.8)	259 (78.0)	674 (74.7)
Nervous system disorders (Continued)			
Masked facies	1 (0.2)	3 (0.9)	4 (0.4)
Memory impairment	0 (0.0)	1 (0.3)	1 (0.1)
Mental impairment	2 (0.4)	0 (0.0)	2 (0.2)
Muscle contractions involuntary	0 (0.0)	1 (0.3)	1 (0.1)
Myoclonus	3 (0.5)	0 (0.0)	3 (0.3)
Nerve compression	0 (0.0)	1 (0.3)	1 (0.1)
Nerve root compression	0 (0.0)	1 (0.3)	1 (0.1)
Neuropathy peripheral	0 (0.0)	1 (0.3)	1 (0.1)
Pallanaesthesia	2 (0.4)	0 (0.0)	2 (0.2)
Paraesthesia	1 (0.2)	1 (0.3)	2 (0.2)
Parkinson's disease	1 (0.2)	1 (0.3)	2 (0.2)
Parkinsonian gait	0 (0.0)	1 (0.3)	1 (0.1)
Parkinsonism	5 (0.9)	2 (0.6)	7 (0.8)

Data Source: Listing 16.2.3.1, Listing 16.2.7.1
A treatment-emergent sign or symptom (TESS) is defined as an adverse event that either (1) begins on or after the date of the first dose of study drug of E2020-G000-328 [up to 30 days after date of last dose of study drug of E2020-G000-328] or (2) increases in severity during the treatment period.
Subjects are counted only once per treatment in each row.
Number of subjects in Safety Population is used as the denominator for computing percentages.

tad_ae.sas 25MAY2010 22:15

Table 14.3.1.4
Summary of Treatment-Emergent Signs or Symptoms by Body System and Preferred Term
Safety Population

Body System<br>Preferred Term	Lead-in Treatment Group<br>Donepezil SR 23 mg<br>n (%)	Lead-in Treatment Group<br>Donepezil IR 10 mg<br>n (%)	Total<br>n (%)
Number of Subjects	570	332	902
Number of Subjects with at Least One Treatment-Emergent Sign or Symptom	415 (72.8)	259 (78.0)	674 (74.7)
Nervous system disorders (Continued)			
Peripheral sensory neuropathy	1 (0.2)	0 (0.0)	1 (0.1)
Presyncope	2 (0.4)	0 (0.0)	2 (0.2)
Psychomotor hyperactivity	2 (0.4)	3 (0.9)	5 (0.6)
Radiculopathy	0 (0.0)	1 (0.3)	1 (0.1)
Repetitive speech	0 (0.0)	1 (0.3)	1 (0.1)
Restless legs syndrome	1 (0.2)	0 (0.0)	1 (0.1)
Sciatica	0 (0.0)	1 (0.3)	1 (0.1)
Sleep phase rhythm disturbance	1 (0.2)	0 (0.0)	1 (0.1)
Somnolence	9 (1.6)	6 (1.8)	15 (1.7)
Speech disorder	6 (1.1)	2 (0.6)	8 (0.9)
Status epilepticus	0 (0.0)	1 (0.3)	1 (0.1)
Syncope	19 (3.3)	9 (2.7)	28 (3.1)
Syncope vasovagal	1 (0.2)	1 (0.3)	2 (0.2)

Data Source: Listing 16.2.3.1, Listing 16.2.7.1
A treatment-emergent sign or symptom (TESS) is defined as an adverse event that either (1) begins on or after the date of the first dose of study drug of E2020-G000-328 [up to 30 days after date of last dose of study drug of E2020-G000-328] or (2) increases in severity during the treatment period.
Subjects are counted only once per treatment in each row.
Number of subjects in Safety Population is used as the denominator for computing percentages.

Table 14.3.1.4
Summary of Treatment-Emergent Signs or Symptoms by Body System and Preferred Term
Safety Population

Body System<br>Preferred Term	Lead-in Treatment Group<br>Donepezil SR 23 mg<br>n (%)	 Donepezil IR 10 mg<br>n (%)	Total<br>n (%)
Number of Subjects	570	332	902
Number of Subjects with at Least One Treatment-Emergent Sign or Symptom	415 (72.8)	259 (78.0)	674 (74.7)
Nervous system disorders (Continued)			
Transient ischaemic attack	1 (0.2)	4 (1.2)	5 (0.6)
Tremor	3 (0.5)	6 (1.8)	9 (1.0)
Unresponsive to stimuli	2 (0.4)	0 (0.0)	2 (0.2)
Psychiatric disorders	148 (26.0)	92 (27.7)	240 (26.6)
Abnormal behaviour	2 (0.4)	2 (0.6)	4 (0.4)
Abnormal dreams	1 (0.2)	3 (0.9)	4 (0.4)
Acute psychosis	1 (0.2)	0 (0.0)	1 (0.1)
Affect lability	1 (0.2)	1 (0.3)	2 (0.2)
Aggression	29 (5.1)	23 (6.9)	52 (5.8)
Agitation	33 (5.8)	28 (8.4)	61 (6.8)
Anger	1 (0.2)	1 (0.3)	2 (0.2)
Anxiety	12 (2.1)	2 (0.6)	14 (1.6)

Data Source: Listing 16.2.3.1, Listing 16.2.7.1
A treatment-emergent sign or symptom (TESS) is defined as an adverse event that either (1) begins on or after the date of the first dose of study drug of E2020-G000-328 [up to 30 days after date of last dose of study drug of E2020-G000-328] or (2) increases in severity during the treatment period.
Subjects are counted only once per treatment in each row.
Number of subjects in Safety Population is used as the denominator for computing percentages.

tad_ae.sas 25MAY2010 22:15

Table 14.3.1.4
Summary of Treatment-Emergent Signs or Symptoms by Body System and Preferred Term
Safety Population

Body System<br>Preferred Term	Lead-in Treatment Group<br>Donepezil SR 23 mg<br>n (%)	 Donepezil IR 10 mg<br>n (%)	Total<br>n (%)
Number of Subjects	570	332	902
Number of Subjects with at Least One Treatment-Emergent Sign or Symptom	415 (72.8)	259 (78.0)	674 (74.7)
Psychiatric disorders (Continued)			
Anxiety disorder	1 (0.2)	0 (0.0)	1 (0.1)
Apathy	2 (0.4)	1 (0.3)	3 (0.3)
Behavioural and psychiatric symptoms of dementia	1 (0.2)	0 (0.0)	1 (0.1)
Bruxism	1 (0.2)	0 (0.0)	1 (0.1)
Communication disorder	1 (0.2)	1 (0.3)	2 (0.2)
Conduct disorder	1 (0.2)	0 (0.0)	1 (0.1)
Confusional state	8 (1.4)	6 (1.8)	14 (1.6)
Delirium	5 (0.9)	0 (0.0)	5 (0.6)
Delusion	7 (1.2)	4 (1.2)	11 (1.2)
Delusional disorder, unspecified type	0 (0.0)	1 (0.3)	1 (0.1)
Depressed mood	1 (0.2)	0 (0.0)	1 (0.1)
Depression	15 (2.6)	12 (3.6)	27 (3.0)
Dermatillomania	1 (0.2)	0 (0.0)	1 (0.1)

Data Source: Listing 16.2.3.1, Listing 16.2.7.1
A treatment-emergent sign or symptom (TESS) is defined as an adverse event that either (1) begins on or after the date of the first dose of study drug of E2020-G000-328 [up to 30 days after date of last dose of study drug of E2020-G000-328] or (2) increases in severity during the treatment period.
Subjects are counted only once per treatment in each row.
Number of subjects in Safety Population is used as the denominator for computing percentages.

Table 14.3.1.4
Summary of Treatment-Emergent Signs or Symptoms by Body System and Preferred Term
Safety Population

Body System<br>Preferred Term	Lead-in Treatment Group<br>Donepezil SR 23 mg<br>n (%)	 Donepezil IR 10 mg<br>n (%)	Total<br>n (%)
Number of Subjects	570	332	902
Number of Subjects with at Least One Treatment-Emergent Sign or Symptom	415 (72.8)	259 (78.0)	674 (74.7)
Psychiatric disorders (Continued)			
Disinhibition	1 (0.2)	4 (1.2)	5 (0.6)
Disorientation	1 (0.2)	1 (0.3)	2 (0.2)
Distractibility	1 (0.2)	0 (0.0)	1 (0.1)
Euphoric mood	1 (0.2)	0 (0.0)	1 (0.1)
Excessive masturbation	0 (0.0)	1 (0.3)	1 (0.1)
Expressive language disorder	2 (0.4)	0 (0.0)	2 (0.2)
Hallucination	9 (1.6)	3 (0.9)	12 (1.3)
Hallucination, auditory	1 (0.2)	2 (0.6)	3 (0.3)
Hallucination, visual	10 (1.8)	5 (1.5)	15 (1.7)
Hostility	0 (0.0)	1 (0.3)	1 (0.1)
Impaired self-care	1 (0.2)	1 (0.3)	2 (0.2)
Impatience	0 (0.0)	1 (0.3)	1 (0.1)
Impulsive behaviour	0 (0.0)	2 (0.6)	2 (0.2)

Data Source: Listing 16.2.3.1, Listing 16.2.7.1
A treatment-emergent sign or symptom (TESS) is defined as an adverse event that either (1) begins on or after the date of the first dose of study drug of E2020-G000-328 [up to 30 days after date of last dose of study drug of E2020-G000-328] or (2) increases in severity during the treatment period.
Subjects are counted only once per treatment in each row.
Number of subjects in Safety Population is used as the denominator for computing percentages.

tad_ae.sas 25MAY2010 22:15

Table 14.3.1.4
Summary of Treatment-Emergent Signs or Symptoms by Body System and Preferred Term
Safety Population

Body System<br>Preferred Term	Lead-in Treatment Group<br>Donepezil SR 23 mg<br>n (%)	Lead-in Treatment Group<br>Donepezil IR 10 mg<br>n (%)	Total<br>n (%)
Number of Subjects	570	332	902
Number of Subjects with at Least One Treatment-Emergent Sign or Symptom	415 (72.8)	259 (78.0)	674 (74.7)
Psychiatric disorders (Continued)			
Insomnia	18 (3.2)	15 (4.5)	33 (3.7)
Libido increased	1 (0.2)	0 (0.0)	1 (0.1)
Major depression	1 (0.2)	1 (0.3)	2 (0.2)
Mania	1 (0.2)	0 (0.0)	1 (0.1)
Mental status changes	0 (0.0)	5 (1.5)	5 (0.6)
Mood swings	0 (0.0)	1 (0.3)	1 (0.1)
Negativism	1 (0.2)	0 (0.0)	1 (0.1)
Nightmare	3 (0.5)	1 (0.3)	4 (0.4)
Paranoia	1 (0.2)	0 (0.0)	1 (0.1)
Personality disorder	1 (0.2)	0 (0.0)	1 (0.1)
Poriomania	2 (0.4)	0 (0.0)	2 (0.2)
Poverty of speech	1 (0.2)	1 (0.3)	2 (0.2)
Psychotic disorder	1 (0.2)	1 (0.3)	2 (0.2)

Data Source: Listing 16.2.3.1, Listing 16.2.7.1
A treatment-emergent sign or symptom (TESS) is defined as an adverse event that either (1) begins on or after the date of the first dose of study drug of E2020-G000-328 [up to 30 days after date of last dose of study drug of E2020-G000-328] or (2) increases in severity during the treatment period.
Subjects are counted only once per treatment in each row.
Number of subjects in Safety Population is used as the denominator for computing percentages.

tad_ae.sas 25MAY2010 22:15

Table 14.3.1.4
Summary of Treatment-Emergent Signs or Symptoms by Body System and Preferred Term
Safety Population

Body System<br>Preferred Term	Lead-in Treatment Group<br>Donepezil SR 23 mg<br>n (%)	 Donepezil IR 10 mg<br>n (%)	Total<br>n (%)
Number of Subjects	570	332	902
Number of Subjects with at Least One Treatment-Emergent Sign or Symptom	415 (72.8)	259 (78.0)	674 (74.7)
Psychiatric disorders (Continued)			
Restlessness	9 (1.6)	6 (1.8)	15 (1.7)
Sleep disorder	3 (0.5)	2 (0.6)	5 (0.6)
Social avoidant behaviour	1 (0.2)	0 (0.0)	1 (0.1)
Soliloquy	1 (0.2)	1 (0.3)	2 (0.2)
Suicidal ideation	1 (0.2)	0 (0.0)	1 (0.1)
Suspiciousness	1 (0.2)	0 (0.0)	1 (0.1)
Tic	2 (0.4)	0 (0.0)	2 (0.2)
Renal and urinary disorders	36 (6.3)	33 (9.9)	69 (7.6)
Calculus bladder	1 (0.2)	0 (0.0)	1 (0.1)
Chromaturia	0 (0.0)	1 (0.3)	1 (0.1)
Dysuria	1 (0.2)	3 (0.9)	4 (0.4)
Enuresis	1 (0.2)	0 (0.0)	1 (0.1)

Data Source: Listing 16.2.3.1, Listing 16.2.7.1
A treatment-emergent sign or symptom (TESS) is defined as an adverse event that either (1) begins on or after the date of the first dose of study drug of E2020-G000-328 [up to 30 days after date of last dose of study drug of E2020-G000-328] or (2) increases in severity during the treatment period.
Subjects are counted only once per treatment in each row.
Number of subjects in Safety Population is used as the denominator for computing percentages.

tad_ae.sas 25MAY2010 22:15

Table 14.3.1.4
Summary of Treatment-Emergent Signs or Symptoms by Body System and Preferred Term
Safety Population

Body System<br>Preferred Term	Lead-in Treatment Group<br>Donepezil SR 23 mg<br>n (%)	 Donepezil IR 10 mg<br>n (%)	Total<br>n (%)
Number of Subjects	570	332	902
Number of Subjects with at Least One Treatment-Emergent Sign or Symptom	415 (72.8)	259 (78.0)	674 (74.7)
Renal and urinary disorders (Continued)			
Haematuria	7 (1.2)	7 (2.1)	14 (1.6)
Hypertonic bladder	1 (0.2)	0 (0.0)	1 (0.1)
Micturition urgency	1 (0.2)	1 (0.3)	2 (0.2)
Nephrolithiasis	1 (0.2)	0 (0.0)	1 (0.1)
Neurogenic bladder	0 (0.0)	1 (0.3)	1 (0.1)
Nocturia	0 (0.0)	1 (0.3)	1 (0.1)
Pollakiuria	4 (0.7)	5 (1.5)	9 (1.0)
Polyuria	0 (0.0)	1 (0.3)	1 (0.1)
Proteinuria	1 (0.2)	1 (0.3)	2 (0.2)
Pyuria	1 (0.2)	0 (0.0)	1 (0.1)
Renal cyst	1 (0.2)	1 (0.3)	2 (0.2)
Renal failure	3 (0.5)	0 (0.0)	3 (0.3)
Renal failure acute	1 (0.2)	2 (0.6)	3 (0.3)

Data Source: Listing 16.2.3.1, Listing 16.2.7.1
A treatment-emergent sign or symptom (TESS) is defined as an adverse event that either (1) begins on or after the date of the first dose of study drug of E2020-G000-328 [up to 30 days after date of last dose of study drug of E2020-G000-328] or (2) increases in severity during the treatment period.
Subjects are counted only once per treatment in each row.
Number of subjects in Safety Population is used as the denominator for computing percentages.

tad_ae.sas 25MAY2010 22:15

Table 14.3.1.4
Summary of Treatment-Emergent Signs or Symptoms by Body System and Preferred Term
Safety Population

Body System<br>Preferred Term	Lead-in Treatment Group<br>Donepezil SR 23 mg<br>n (%)	 Donepezil IR 10 mg<br>n (%)	Total<br>n (%)
Number of Subjects	570	332	902
Number of Subjects with at Least One Treatment-Emergent Sign or Symptom	415 (72.8)	259 (78.0)	674 (74.7)
Renal and urinary disorders (Continued)			
Renal failure chronic	2 (0.4)	0 (0.0)	2 (0.2)
Renal impairment	0 (0.0)	2 (0.6)	2 (0.2)
Urinary incontinence	12 (2.1)	11 (3.3)	23 (2.5)
Urinary retention	1 (0.2)	0 (0.0)	1 (0.1)
Reproductive system and breast disorders	8 (1.4)	9 (2.7)	17 (1.9)
Atrophic vulvovaginitis	0 (0.0)	1 (0.3)	1 (0.1)
Benign prostatic hyperplasia	3 (0.5)	5 (1.5)	8 (0.9)
Haematospermia	0 (0.0)	1 (0.3)	1 (0.1)
Metrorrhagia	0 (0.0)	2 (0.6)	2 (0.2)
Prostatism	1 (0.2)	0 (0.0)	1 (0.1)
Prostatitis	1 (0.2)	0 (0.0)	1 (0.1)
Prostatomegaly	2 (0.4)	0 (0.0)	2 (0.2)
Vaginal lesion	1 (0.2)	0 (0.0)	1 (0.1)

Data Source: Listing 16.2.3.1, Listing 16.2.7.1
A treatment-emergent sign or symptom (TESS) is defined as an adverse event that either (1) begins on or after the date of the first dose of study drug of E2020-G000-328 [up to 30 days after date of last dose of study drug of E2020-G000-328] or (2) increases in severity during the treatment period.
Subjects are counted only once per treatment in each row.
Number of subjects in Safety Population is used as the denominator for computing percentages.

tad_ae.sas 25MAY2010 22:15

Table 14.3.1.4
Summary of Treatment-Emergent Signs or Symptoms by Body System and Preferred Term
Safety Population

	Lead-in Treatment Group		
Body System<br>Preferred Term	Donepezil SR 23 mg<br>n (%)	Donepezil IR 10 mg<br>n (%)	Total<br>n (%)
Number of Subjects	570	332	902
Number of Subjects with at Least One Treatment-Emergent Sign or Symptom	415 (72.8)	259 (78.0)	674 (74.7)
Respiratory, thoracic and mediastinal disorders	39 (6.8)	16 (4.8)	55 (6.1)
Asthma	2 (0.4)	0 (0.0)	2 (0.2)
Atelectasis	1 (0.2)	0 (0.0)	1 (0.1)
Bronchial obstruction	1 (0.2)	0 (0.0)	1 (0.1)
Choking	1 (0.2)	0 (0.0)	1 (0.1)
Chronic obstructive pulmonary disease	1 (0.2)	1 (0.3)	2 (0.2)
Cough	7 (1.2)	7 (2.1)	14 (1.6)
Dyspnoea	3 (0.5)	0 (0.0)	3 (0.3)
Dyspnoea exertional	1 (0.2)	0 (0.0)	1 (0.1)
Epistaxis	2 (0.4)	0 (0.0)	2 (0.2)
Lung disorder	2 (0.4)	0 (0.0)	2 (0.2)
Lung infiltration	1 (0.2)	0 (0.0)	1 (0.1)
Nasal congestion	4 (0.7)	0 (0.0)	4 (0.4)
Oropharyngeal pain	2 (0.4)	0 (0.0)	2 (0.2)

Data Source: Listing 16.2.3.1, Listing 16.2.7.1
A treatment-emergent sign or symptom (TESS) is defined as an adverse event that either (1) begins on or after the date of the first dose of study drug of E2020-G000-328 [up to 30 days after date of last dose of study drug of E2020-G000-328] or (2) increases in severity during the treatment period.
Subjects are counted only once per treatment in each row.
Number of subjects in Safety Population is used as the denominator for computing percentages.

Table 14.3.1.4
Summary of Treatment-Emergent Signs or Symptoms by Body System and Preferred Term
Safety Population

Body System<br>Preferred Term	Lead-in Treatment Group<br>Donepezil SR 23 mg<br>n (%)	 Donepezil IR 10 mg<br>n (%)	Total<br>n (%)
Number of Subjects	570	332	902
Number of Subjects with at Least One Treatment-Emergent Sign or Symptom	415 (72.8)	259 (78.0)	674 (74.7)
Respiratory, thoracic and mediastinal disorders (Continued)			
Paranasal sinus hypersecretion	0 (0.0)	1 (0.3)	1 (0.1)
Pleural effusion	1 (0.2)	0 (0.0)	1 (0.1)
Pleural fibrosis	1 (0.2)	0 (0.0)	1 (0.1)
Pneumonitis	1 (0.2)	0 (0.0)	1 (0.1)
Pneumothorax	1 (0.2)	0 (0.0)	1 (0.1)
Postnasal drip	2 (0.4)	1 (0.3)	3 (0.3)
Productive cough	3 (0.5)	0 (0.0)	3 (0.3)
Pulmonary embolism	0 (0.0)	2 (0.6)	2 (0.2)
Rhinitis allergic	0 (0.0)	1 (0.3)	1 (0.1)
Rhinorrhoea	4 (0.7)	1 (0.3)	5 (0.6)
Rhonchi	1 (0.2)	0 (0.0)	1 (0.1)
Sinus congestion	0 (0.0)	1 (0.3)	1 (0.1)
Upper respiratory tract congestion	1 (0.2)	0 (0.0)	1 (0.1)
Wheezing	2 (0.4)	2 (0.6)	4 (0.4)

Data Source: Listing 16.2.3.1, Listing 16.2.7.1
A treatment-emergent sign or symptom (TESS) is defined as an adverse event that either (1) begins on or after the date of the first dose of study drug of E2020-G000-328 [up to 30 days after date of last dose of study drug of E2020-G000-328] or (2) increases in severity during the treatment period.
Subjects are counted only once per treatment in each row.
Number of subjects in Safety Population is used as the denominator for computing percentages.

tad_ae.sas 25MAY2010 22:15

Table 14.3.1.4
Summary of Treatment-Emergent Signs or Symptoms by Body System and Preferred Term
Safety Population

	Lead-in Treatment Group		
Body System<br>Preferred Term	Donepezil SR 23 mg<br>n (%)	Donepezil IR 10 mg<br>n (%)	Total<br>n (%)
Number of Subjects	570	332	902
Number of Subjects with at Least One Treatment-Emergent Sign or Symptom	415 (72.8)	259 (78.0)	674 (74.7)
Skin and subcutaneous tissue disorders	43 (7.5)	16 (4.8)	59 (6.5)
Acne	0 (0.0)	1 (0.3)	1 (0.1)
Actinic keratosis	1 (0.2)	0 (0.0)	1 (0.1)
Alopecia	3 (0.5)	0 (0.0)	3 (0.3)
Blister	0 (0.0)	1 (0.3)	1 (0.1)
Dandruff	1 (0.2)	0 (0.0)	1 (0.1)
Decubitus ulcer	3 (0.5)	0 (0.0)	3 (0.3)
Dermatitis	1 (0.2)	3 (0.9)	4 (0.4)
Dermatitis allergic	2 (0.4)	0 (0.0)	2 (0.2)
Dermatitis contact	1 (0.2)	1 (0.3)	2 (0.2)
Dermatitis diaper	1 (0.2)	0 (0.0)	1 (0.1)
Dry skin	2 (0.4)	0 (0.0)	2 (0.2)
Ecchymosis	1 (0.2)	3 (0.9)	4 (0.4)
Eczema	3 (0.5)	0 (0.0)	3 (0.3)

Data Source: Listing 16.2.3.1, Listing 16.2.7.1
A treatment-emergent sign or symptom (TESS) is defined as an adverse event that either (1) begins on or after the date of the first dose of study drug of E2020-G000-328 [up to 30 days after date of last dose of study drug of E2020-G000-328] or (2) increases in severity during the treatment period.
Subjects are counted only once per treatment in each row.
Number of subjects in Safety Population is used as the denominator for computing percentages.

tad_ae.sas 25MAY2010 22:15

Table 14.3.1.4
Summary of Treatment-Emergent Signs or Symptoms by Body System and Preferred Term
Safety Population

	Lead-in Treatment Group		
Body System<br>Preferred Term	Donepezil SR 23 mg<br>n (%)	Donepezil IR 10 mg<br>n (%)	Total<br>n (%)
Number of Subjects	570	332	902
Number of Subjects with at Least One Treatment-Emergent Sign or Symptom	415 (72.8)	259 (78.0)	674 (74.7)
Skin and subcutaneous tissue disorders (Continued)			
Erythema	2 (0.4)	0 (0.0)	2 (0.2)
Erythema multiforme	1 (0.2)	0 (0.0)	1 (0.1)
Hyperkeratosis	2 (0.4)	1 (0.3)	3 (0.3)
Night sweats	1 (0.2)	0 (0.0)	1 (0.1)
Pruritus	1 (0.2)	0 (0.0)	1 (0.1)
Rash	3 (0.5)	4 (1.2)	7 (0.8)
Rash generalised	1 (0.2)	0 (0.0)	1 (0.1)
Rash papular	1 (0.2)	0 (0.0)	1 (0.1)
Rash pruritic	1 (0.2)	1 (0.3)	2 (0.2)
Rosacea	1 (0.2)	0 (0.0)	1 (0.1)
Seborrhoeic dermatitis	1 (0.2)	0 (0.0)	1 (0.1)
Skin discolouration	1 (0.2)	0 (0.0)	1 (0.1)
Skin disorder	1 (0.2)	0 (0.0)	1 (0.1)

Data Source: Listing 16.2.3.1, Listing 16.2.7.1
A treatment-emergent sign or symptom (TESS) is defined as an adverse event that either (1) begins on or after the date of the first dose of study drug of E2020-G000-328 [up to 30 days after date of last dose of study drug of E2020-G000-328] or (2) increases in severity during the treatment period.
Subjects are counted only once per treatment in each row.
Number of subjects in Safety Population is used as the denominator for computing percentages.

tad_ae.sas 25MAY2010 22:15

Table 14.3.1.4
Summary of Treatment-Emergent Signs or Symptoms by Body System and Preferred Term
Safety Population

Body System<br>Preferred Term	Lead-in Treatment Group<br>Donepezil SR 23 mg<br>n (%)	 Donepezil IR 10 mg<br>n (%)	Total<br>n (%)
Number of Subjects	570	332	902
Number of Subjects with at Least One Treatment-Emergent Sign or Symptom	415 (72.8)	259 (78.0)	674 (74.7)
Skin and subcutaneous tissue disorders (Continued)			
Skin erosion	1 (0.2)	0 (0.0)	1 (0.1)
Skin irritation	0 (0.0)	1 (0.3)	1 (0.1)
Skin lesion	6 (1.1)	1 (0.3)	7 (0.8)
Skin odour abnormal	1 (0.2)	0 (0.0)	1 (0.1)
Swelling face	1 (0.2)	0 (0.0)	1 (0.1)
Social circumstances	3 (0.5)	1 (0.3)	4 (0.4)
Activities of daily living impaired	2 (0.4)	1 (0.3)	3 (0.3)
Verbal abuse	1 (0.2)	0 (0.0)	1 (0.1)
Vascular disorders	30 (5.3)	22 (6.6)	52 (5.8)
Aortic stenosis	1 (0.2)	0 (0.0)	1 (0.1)
Capillary fragility	0 (0.0)	1 (0.3)	1 (0.1)

Data Source: Listing 16.2.3.1, Listing 16.2.7.1
A treatment-emergent sign or symptom (TESS) is defined as an adverse event that either (1) begins on or after the date of the first dose of study drug of E2020-G000-328 [up to 30 days after date of last dose of study drug of E2020-G000-328] or (2) increases in severity during the treatment period.
Subjects are counted only once per treatment in each row.
Number of subjects in Safety Population is used as the denominator for computing percentages.

tad_ae.sas 25MAY2010 22:15

Table 14.3.1.4
Summary of Treatment-Emergent Signs or Symptoms by Body System and Preferred Term
Safety Population

Body System<br>Preferred Term	Lead-in Treatment Group<br>Donepezil SR 23 mg<br>n (%)	Lead-in Treatment Group<br>Donepezil IR 10 mg<br>n (%)	Total<br>n (%)
Number of Subjects	570	332	902
Number of Subjects with at Least One Treatment-Emergent Sign or Symptom	415 (72.8)	259 (78.0)	674 (74.7)
Vascular disorders (Continued)			
Deep vein thrombosis	1 (0.2)	0 (0.0)	1 (0.1)
Flushing	0 (0.0)	1 (0.3)	1 (0.1)
Haematoma	1 (0.2)	0 (0.0)	1 (0.1)
Hot flush	2 (0.4)	1 (0.3)	3 (0.3)
Hypertension	18 (3.2)	10 (3.0)	28 (3.1)
Hypotension	5 (0.9)	4 (1.2)	9 (1.0)
Orthostatic hypotension	2 (0.4)	4 (1.2)	6 (0.7)
Phlebitis	1 (0.2)	0 (0.0)	1 (0.1)
Varicose vein	0 (0.0)	2 (0.6)	2 (0.2)

Data Source: Listing 16.2.3.1, Listing 16.2.7.1
A treatment-emergent sign or symptom (TESS) is defined as an adverse event that either (1) begins on or after the date of the first dose of study drug of E2020-G000-328 [up to 30 days after date of last dose of study drug of E2020-G000-328] or (2) increases in severity during the treatment period.
Subjects are counted only once per treatment in each row.
Number of subjects in Safety Population is used as the denominator for computing percentages.
