# Supplementary material for: Long-term safety and tolerability of donepezil 23 mg in patients with moderate to severe Alzheimer’s disease
Source: BMC Res Notes. 2012 Jun 8;5:283. doi: 10.1186/1756-0500-5-283 (PMC3493328; doi:10.1186/1756-0500-5-283)
Supplement: Additional file 3 — Summary of Serious Treatment-Emergent Signs or Symptoms by Body System and Preferred Term (Safety Population). [file 1756-0500-5-283-S3.pdf]

Table 14.3.2.2.2
Summary of Serious Treatment-Emergent Signs or Symptoms by Body System and Preferred Term
Safety Population

	Lead-in Treatment Group		
Body System<br>Preferred Term	Donepezil SR 23 mg<br>n (%)	Donepezil IR 10 mg<br>n (%)	Total<br>n (%)
Number of Subjects	570	332	902
Number of subjects with serious treatment-emergent signs or symptoms	80 (14.0)	56 (16.9)	136 (15.1)
Blood and lymphatic system disorders	1 (0.2)	0 (0.0)	1 (0.1)
Anaemia	1 (0.2)	0 (0.0)	1 (0.1)
Cardiac disorders	7 (1.2)	5 (1.5)	12 (1.3)
Acute myocardial infarction	0 (0.0)	1 (0.3)	1 (0.1)
Angina pectoris	1 (0.2)	0 (0.0)	1 (0.1)
Arrhythmia supraventricular	0 (0.0)	1 (0.3)	1 (0.1)
Atrial fibrillation	0 (0.0)	2 (0.6)	2 (0.2)
Bradycardia	2 (0.4)	0 (0.0)	2 (0.2)
Cardiac failure	1 (0.2)	1 (0.3)	2 (0.2)
Cardio-respiratory arrest	1 (0.2)	0 (0.0)	1 (0.1)

Data Source: Listing 16.2.3.1, Listing 16.2.7.1
A treatment-emergent sign or symptom (TESS) is defined as an adverse event that either (1) begins on or after the date of the first dose of study drug of E2020-G000-328 [up to 30 days after date of last dose of study drug of E2020-G000-328] or (2) increases in severity during the treatment period.
Subjects are counted only once per treatment in each row.
Number of subjects in Safety Population is used as the denominator for computing percentages.

tad_ae.sas 25MAY2010 22:15

Table 14.3.2.2.2
Summary of Serious Treatment-Emergent Signs or Symptoms by Body System and Preferred Term
Safety Population

Body System<br>Preferred Term	Lead-in Treatment Group<br>Donepezil SR 23 mg<br>n (%)	 Donepezil IR 10 mg<br>n (%)	Total<br>n (%)
Number of Subjects	570	332	902
Number of subjects with serious treatment-emergent signs or symptoms	80 (14.0)	56 (16.9)	136 (15.1)
Cardiac disorders (Continued)			
Myocardial infarction	2 (0.4)	0 (0.0)	2 (0.2)
Ventricular tachycardia	1 (0.2)	0 (0.0)	1 (0.1)
Congenital, familial and genetic disorders	1 (0.2)	1 (0.3)	2 (0.2)
Encephalocele	0 (0.0)	1 (0.3)	1 (0.1)
Hydrocele	1 (0.2)	0 (0.0)	1 (0.1)
Meningocele	0 (0.0)	1 (0.3)	1 (0.1)
Gastrointestinal disorders	8 (1.4)	5 (1.5)	13 (1.4)
Abdominal pain	0 (0.0)	1 (0.3)	1 (0.1)
Diarrhoea	1 (0.2)	1 (0.3)	2 (0.2)
Duodenal ulcer perforation	1 (0.2)	0 (0.0)	1 (0.1)

Data Source: Listing 16.2.3.1, Listing 16.2.7.1
A treatment-emergent sign or symptom (TESS) is defined as an adverse event that either (1) begins on or after the date of the first dose of study drug of E2020-G000-328 [up to 30 days after date of last dose of study drug of E2020-G000-328] or (2) increases in severity during the treatment period.
Subjects are counted only once per treatment in each row.
Number of subjects in Safety Population is used as the denominator for computing percentages.

Table 14.3.2.2.2
Summary of Serious Treatment-Emergent Signs or Symptoms by Body System and Preferred Term
Safety Population

Body System<br>Preferred Term	Lead-in Treatment Group<br>Donepezil SR 23 mg<br>n (%)	Lead-in Treatment Group<br>Donepezil IR 10 mg<br>n (%)	Total<br>n (%)
Number of Subjects	570	332	902
Number of subjects with serious treatment-emergent signs or symptoms	80 (14.0)	56 (16.9)	136 (15.1)
Gastrointestinal disorders (Continued)			
Gastric ulcer	0 (0.0)	1 (0.3)	1 (0.1)
Gastrointestinal haemorrhage	1 (0.2)	0 (0.0)	1 (0.1)
Haematemesis	0 (0.0)	2 (0.6)	2 (0.2)
Haematochezia	1 (0.2)	0 (0.0)	1 (0.1)
Ileus paralytic	1 (0.2)	0 (0.0)	1 (0.1)
Oesophageal ulcer	1 (0.2)	0 (0.0)	1 (0.1)
Pancreatic mass	1 (0.2)	0 (0.0)	1 (0.1)
Upper gastrointestinal haemorrhage	1 (0.2)	0 (0.0)	1 (0.1)
Vomiting	0 (0.0)	1 (0.3)	1 (0.1)
General disorders and administration site conditions	6 (1.1)	3 (0.9)	9 (1.0)
Asthenia	1 (0.2)	1 (0.3)	2 (0.2)

Data Source: Listing 16.2.3.1, Listing 16.2.7.1
A treatment-emergent sign or symptom (TESS) is defined as an adverse event that either (1) begins on or after the date of the first dose of study drug of E2020-G000-328 [up to 30 days after date of last dose of study drug of E2020-G000-328] or (2) increases in severity during the treatment period.
Subjects are counted only once per treatment in each row.
Number of subjects in Safety Population is used as the denominator for computing percentages.

tad_ae.sas 25MAY2010 22:15

Table 14.3.2.2.2
Summary of Serious Treatment-Emergent Signs or Symptoms by Body System and Preferred Term
Safety Population

Body System<br>Preferred Term	Lead-in Treatment Group<br>Donepezil SR 23 mg<br>n (%)	Lead-in Treatment Group<br>Donepezil IR 10 mg<br>n (%)	Total<br>n (%)
Number of Subjects	570	332	902
Number of subjects with serious treatment-emergent signs or symptoms	80 (14.0)	56 (16.9)	136 (15.1)
General disorders and administration site conditions (Continued)			
Chest pain	1 (0.2)	0 (0.0)	1 (0.1)
Death	2 (0.4)	0 (0.0)	2 (0.2)
Gait disturbance	1 (0.2)	0 (0.0)	1 (0.1)
Multi-organ failure	0 (0.0)	1 (0.3)	1 (0.1)
Non-cardiac chest pain	1 (0.2)	0 (0.0)	1 (0.1)
Sudden death	0 (0.0)	1 (0.3)	1 (0.1)
Hepatobiliary disorders	0 (0.0)	2 (0.6)	2 (0.2)
Cholecystitis acute	0 (0.0)	1 (0.3)	1 (0.1)
Hepatic cirrhosis	0 (0.0)	1 (0.3)	1 (0.1)

Data Source: Listing 16.2.3.1, Listing 16.2.7.1
A treatment-emergent sign or symptom (TESS) is defined as an adverse event that either (1) begins on or after the date of the first dose of study drug of E2020-G000-328 [up to 30 days after date of last dose of study drug of E2020-G000-328] or (2) increases in severity during the treatment period.
Subjects are counted only once per treatment in each row.
Number of subjects in Safety Population is used as the denominator for computing percentages.

tad_ae.sas 25MAY2010 22:15

Table 14.3.2.2.2
Summary of Serious Treatment-Emergent Signs or Symptoms by Body System and Preferred Term
Safety Population

	Lead-in Treatment Group		
Body System<br>Preferred Term	Donepezil SR 23 mg<br>n (%)	Donepezil IR 10 mg<br>n (%)	Total<br>n (%)
Number of Subjects	570	332	902
Number of subjects with serious treatment-emergent signs or symptoms	80 (14.0)	56 (16.9)	136 (15.1)
Infections and infestations	22 (3.9)	8 (2.4)	30 (3.3)
Abdominal abscess	1 (0.2)	0 (0.0)	1 (0.1)
Bronchitis	0 (0.0)	1 (0.3)	1 (0.1)
Cellulitis staphylococcal	1 (0.2)	0 (0.0)	1 (0.1)
Gastroenteritis	2 (0.4)	0 (0.0)	2 (0.2)
Liver abscess	0 (0.0)	1 (0.3)	1 (0.1)
Lobar pneumonia	0 (0.0)	1 (0.3)	1 (0.1)
Lower respiratory tract infection	2 (0.4)	0 (0.0)	2 (0.2)
Orchitis	1 (0.2)	0 (0.0)	1 (0.1)
Peritonsillar abscess	1 (0.2)	0 (0.0)	1 (0.1)
Pneumonia	6 (1.1)	1 (0.3)	7 (0.8)
Respiratory tract infection	0 (0.0)	1 (0.3)	1 (0.1)
Sepsis	1 (0.2)	0 (0.0)	1 (0.1)

Data Source: Listing 16.2.3.1, Listing 16.2.7.1
A treatment-emergent sign or symptom (TESS) is defined as an adverse event that either (1) begins on or after the date of the first dose of study drug of E2020-G000-328 [up to 30 days after date of last dose of study drug of E2020-G000-328] or (2) increases in severity during the treatment period.
Subjects are counted only once per treatment in each row.
Number of subjects in Safety Population is used as the denominator for computing percentages.

Table 14.3.2.2.2
Summary of Serious Treatment-Emergent Signs or Symptoms by Body System and Preferred Term
Safety Population

Body System<br>Preferred Term	Lead-in Treatment Group<br>Donepezil SR 23 mg<br>n (%)	Lead-in Treatment Group<br>Donepezil IR 10 mg<br>n (%)	Total<br>n (%)
Number of Subjects	570	332	902
Number of subjects with serious treatment-emergent signs or symptoms	80 (14.0)	56 (16.9)	136 (15.1)
Infections and infestations (Continued)			
Tuberculosis	1 (0.2)	0 (0.0)	1 (0.1)
Urinary tract infection	8 (1.4)	3 (0.9)	11 (1.2)
Injury, poisoning and procedural complications	18 (3.2)	9 (2.7)	27 (3.0)
Concussion	1 (0.2)	0 (0.0)	1 (0.1)
Device lead damage	1 (0.2)	0 (0.0)	1 (0.1)
Dislocation of joint prosthesis	0 (0.0)	1 (0.3)	1 (0.1)
Fall	7 (1.2)	2 (0.6)	9 (1.0)
Femoral neck fracture	3 (0.5)	0 (0.0)	3 (0.3)
Femur fracture	2 (0.4)	0 (0.0)	2 (0.2)
Head injury	1 (0.2)	0 (0.0)	1 (0.1)
Hip fracture	2 (0.4)	1 (0.3)	3 (0.3)

Data Source: Listing 16.2.3.1, Listing 16.2.7.1
A treatment-emergent sign or symptom (TESS) is defined as an adverse event that either (1) begins on or after the date of the first dose of study drug of E2020-G000-328 [up to 30 days after date of last dose of study drug of E2020-G000-328] or (2) increases in severity during the treatment period.
Subjects are counted only once per treatment in each row.
Number of subjects in Safety Population is used as the denominator for computing percentages.

Table 14.3.2.2.2
Summary of Serious Treatment-Emergent Signs or Symptoms by Body System and Preferred Term
Safety Population

Body System<br>Preferred Term	Lead-in Treatment Group<br>Donepezil SR 23 mg<br>n (%)	 Donepezil IR 10 mg<br>n (%)	Total<br>n (%)
Number of Subjects	570	332	902
Number of subjects with serious treatment-emergent signs or symptoms	80 (14.0)	56 (16.9)	136 (15.1)
Injury, poisoning and procedural complications (Continued)			
Laceration	1 (0.2)	1 (0.3)	2 (0.2)
Lumbar vertebral fracture	1 (0.2)	0 (0.0)	1 (0.1)
Radius fracture	0 (0.0)	1 (0.3)	1 (0.1)
Road traffic accident	1 (0.2)	0 (0.0)	1 (0.1)
Skin laceration	1 (0.2)	0 (0.0)	1 (0.1)
Subdural haematoma	1 (0.2)	3 (0.9)	4 (0.4)
Subdural haemorrhage	1 (0.2)	0 (0.0)	1 (0.1)
Thoracic vertebral fracture	1 (0.2)	0 (0.0)	1 (0.1)
Upper limb fracture	1 (0.2)	1 (0.3)	2 (0.2)
Investigations	2 (0.4)	1 (0.3)	3 (0.3)
Electrocardiogram QT prolonged	1 (0.2)	0 (0.0)	1 (0.1)

Data Source: Listing 16.2.3.1, Listing 16.2.7.1
A treatment-emergent sign or symptom (TESS) is defined as an adverse event that either (1) begins on or after the date of the first dose of study drug of E2020-G000-328 [up to 30 days after date of last dose of study drug of E2020-G000-328] or (2) increases in severity during the treatment period.
Subjects are counted only once per treatment in each row.
Number of subjects in Safety Population is used as the denominator for computing percentages.

tad_ae.sas 25MAY2010 22:15

Table 14.3.2.2.2
Summary of Serious Treatment-Emergent Signs or Symptoms by Body System and Preferred Term
Safety Population

	Lead-in Treatment Group		
Body System<br>Preferred Term	Donepezil SR 23 mg<br>n (%)	Donepezil IR 10 mg<br>n (%)	Total<br>n (%)
Number of Subjects	570	332	902
Number of subjects with serious treatment-emergent signs or symptoms	80 (14.0)	56 (16.9)	136 (15.1)
Investigations (Continued)			
Lipase increased	0 (0.0)	1 (0.3)	1 (0.1)
Weight decreased	1 (0.2)	0 (0.0)	1 (0.1)
Metabolism and nutrition disorders	3 (0.5)	6 (1.8)	9 (1.0)
Anorexia	0 (0.0)	1 (0.3)	1 (0.1)
Dehydration	2 (0.4)	3 (0.9)	5 (0.6)
Gout	0 (0.0)	1 (0.3)	1 (0.1)
Hypokalaemia	1 (0.2)	0 (0.0)	1 (0.1)
Hyponatraemia	0 (0.0)	2 (0.6)	2 (0.2)
Musculoskeletal and connective tissue disorders	1 (0.2)	1 (0.3)	2 (0.2)
Muscle rigidity	0 (0.0)	1 (0.3)	1 (0.1)
Osteoarthritis	1 (0.2)	0 (0.0)	1 (0.1)

Data Source: Listing 16.2.3.1, Listing 16.2.7.1
A treatment-emergent sign or symptom (TESS) is defined as an adverse event that either (1) begins on or after the date of the first dose of study drug of E2020-G000-328 [up to 30 days after date of last dose of study drug of E2020-G000-328] or (2) increases in severity during the treatment period.
Subjects are counted only once per treatment in each row.
Number of subjects in Safety Population is used as the denominator for computing percentages.

Table 14.3.2.2.2
Summary of Serious Treatment-Emergent Signs or Symptoms by Body System and Preferred Term
Safety Population

	Lead-in Treatment Group		
Body System<br>Preferred Term	Donepezil SR 23 mg<br>n (%)	Donepezil IR 10 mg<br>n (%)	Total<br>n (%)
Number of Subjects	570	332	902
Number of subjects with serious treatment-emergent signs or symptoms	80 (14.0)	56 (16.9)	136 (15.1)
Neoplasms benign, malignant and unspecified (incl cysts and polyps)	6 (1.1)	5 (1.5)	11 (1.2)
Breast cancer	1 (0.2)	0 (0.0)	1 (0.1)
Lung carcinoma cell type unspecified stage IV	1 (0.2)	0 (0.0)	1 (0.1)
Lung neoplasm	1 (0.2)	0 (0.0)	1 (0.1)
Lung neoplasm malignant	1 (0.2)	1 (0.3)	2 (0.2)
Malignant melanoma	0 (0.0)	1 (0.3)	1 (0.1)
Metastases to liver	1 (0.2)	0 (0.0)	1 (0.1)
Metastases to spine	0 (0.0)	1 (0.3)	1 (0.1)
Metastasis	1 (0.2)	0 (0.0)	1 (0.1)
Prostate cancer	0 (0.0)	2 (0.6)	2 (0.2)
Prostate cancer stage II	0 (0.0)	1 (0.3)	1 (0.1)
Rectal cancer	1 (0.2)	0 (0.0)	1 (0.1)

Data Source: Listing 16.2.3.1, Listing 16.2.7.1
A treatment-emergent sign or symptom (TESS) is defined as an adverse event that either (1) begins on or after the date of the first dose of study drug of E2020-G000-328 [up to 30 days after date of last dose of study drug of E2020-G000-328] or (2) increases in severity during the treatment period.
Subjects are counted only once per treatment in each row.
Number of subjects in Safety Population is used as the denominator for computing percentages.

Table 14.3.2.2.2
Summary of Serious Treatment-Emergent Signs or Symptoms by Body System and Preferred Term
Safety Population

Body System<br>Preferred Term	Lead-in Treatment Group<br>Donepezil SR 23 mg<br>n (%)	 Donepezil IR 10 mg<br>n (%)	Total<br>n (%)
Number of Subjects	570	332	902
Number of subjects with serious treatment-emergent signs or symptoms	80 (14.0)	56 (16.9)	136 (15.1)
Nervous system disorders	22 (3.9)	19 (5.7)	41 (4.5)
Bradykinesia	0 (0.0)	1 (0.3)	1 (0.1)
Cerebral atrophy	1 (0.2)	0 (0.0)	1 (0.1)
Cerebral haemorrhage	1 (0.2)	1 (0.3)	2 (0.2)
Cerebral infarction	0 (0.0)	1 (0.3)	1 (0.1)
Cerebrospinal fistula	0 (0.0)	1 (0.3)	1 (0.1)
Cerebrovascular accident	5 (0.9)	2 (0.6)	7 (0.8)
Convulsion	1 (0.2)	0 (0.0)	1 (0.1)
Dementia Alzheimer's type	2 (0.4)	0 (0.0)	2 (0.2)
Dizziness	1 (0.2)	1 (0.3)	2 (0.2)
Encephalopathy	1 (0.2)	0 (0.0)	1 (0.1)
Epilepsy	0 (0.0)	1 (0.3)	1 (0.1)
Headache	0 (0.0)	1 (0.3)	1 (0.1)

Data Source: Listing 16.2.3.1, Listing 16.2.7.1
A treatment-emergent sign or symptom (TESS) is defined as an adverse event that either (1) begins on or after the date of the first dose of study drug of E2020-G000-328 [up to 30 days after date of last dose of study drug of E2020-G000-328] or (2) increases in severity during the treatment period.
Subjects are counted only once per treatment in each row.
Number of subjects in Safety Population is used as the denominator for computing percentages.

tad_ae.sas 25MAY2010 22:15

Table 14.3.2.2.2
Summary of Serious Treatment-Emergent Signs or Symptoms by Body System and Preferred Term
Safety Population

Body System<br>Preferred Term	Lead-in Treatment Group<br>Donepezil SR 23 mg<br>n (%)	 Donepezil IR 10 mg<br>n (%)	Total<br>n (%)
Number of Subjects	570	332	902
Number of subjects with serious treatment-emergent signs or symptoms	80 (14.0)	56 (16.9)	136 (15.1)
Nervous system disorders (Continued)			
Hemiparesis	1 (0.2)	0 (0.0)	1 (0.1)
Hemiplegia	0 (0.0)	1 (0.3)	1 (0.1)
Hydrocephalus	1 (0.2)	0 (0.0)	1 (0.1)
Ischaemic cerebral infarction	0 (0.0)	1 (0.3)	1 (0.1)
Lacunar infarction	0 (0.0)	1 (0.3)	1 (0.1)
Presyncope	1 (0.2)	0 (0.0)	1 (0.1)
Sciatica	0 (0.0)	1 (0.3)	1 (0.1)
Status epilepticus	0 (0.0)	1 (0.3)	1 (0.1)
Syncope	6 (1.1)	6 (1.8)	12 (1.3)
Transient ischaemic attack	1 (0.2)	3 (0.9)	4 (0.4)
Unresponsive to stimuli	1 (0.2)	0 (0.0)	1 (0.1)

Data Source: Listing 16.2.3.1, Listing 16.2.7.1
A treatment-emergent sign or symptom (TESS) is defined as an adverse event that either (1) begins on or after the date of the first dose of study drug of E2020-G000-328 [up to 30 days after date of last dose of study drug of E2020-G000-328] or (2) increases in severity during the treatment period.
Subjects are counted only once per treatment in each row.
Number of subjects in Safety Population is used as the denominator for computing percentages.

Table 14.3.2.2.2
Summary of Serious Treatment-Emergent Signs or Symptoms by Body System and Preferred Term
Safety Population

	Lead-in Treatment Group		
Body System<br>Preferred Term	Donepezil SR 23 mg<br>n (%)	Donepezil IR 10 mg<br>n (%)	Total<br>n (%)
Number of Subjects	570	332	902
Number of subjects with serious treatment-emergent signs or symptoms	80 (14.0)	56 (16.9)	136 (15.1)
Psychiatric disorders	3 (0.5)	10 (3.0)	13 (1.4)
Aggression	1 (0.2)	3 (0.9)	4 (0.4)
Agitation	0 (0.0)	2 (0.6)	2 (0.2)
Anxiety	0 (0.0)	1 (0.3)	1 (0.1)
Hallucination	0 (0.0)	1 (0.3)	1 (0.1)
Insomnia	0 (0.0)	3 (0.9)	3 (0.3)
Mental status changes	0 (0.0)	4 (1.2)	4 (0.4)
Poriomania	1 (0.2)	0 (0.0)	1 (0.1)
Psychotic disorder	0 (0.0)	1 (0.3)	1 (0.1)
Restlessness	1 (0.2)	2 (0.6)	3 (0.3)
Renal and urinary disorders	5 (0.9)	4 (1.2)	9 (1.0)
Calculus bladder	1 (0.2)	0 (0.0)	1 (0.1)

Data Source: Listing 16.2.3.1, Listing 16.2.7.1
A treatment-emergent sign or symptom (TESS) is defined as an adverse event that either (1) begins on or after the date of the first dose of study drug of E2020-G000-328 [up to 30 days after date of last dose of study drug of E2020-G000-328] or (2) increases in severity during the treatment period.
Subjects are counted only once per treatment in each row.
Number of subjects in Safety Population is used as the denominator for computing percentages.

Table 14.3.2.2.2
Summary of Serious Treatment-Emergent Signs or Symptoms by Body System and Preferred Term
Safety Population

	Lead-in Treatment Group		
Body System<br>Preferred Term	Donepezil SR 23 mg<br>n (%)	Donepezil IR 10 mg<br>n (%)	Total<br>n (%)
Number of Subjects	570	332	902
Number of subjects with serious treatment-emergent signs or symptoms	80 (14.0)	56 (16.9)	136 (15.1)
Renal and urinary disorders (Continued)			
Haematuria	1 (0.2)	1 (0.3)	2 (0.2)
Nephrolithiasis	1 (0.2)	0 (0.0)	1 (0.1)
Neurogenic bladder	0 (0.0)	1 (0.3)	1 (0.1)
Renal failure	1 (0.2)	0 (0.0)	1 (0.1)
Renal failure acute	1 (0.2)	2 (0.6)	3 (0.3)
Urinary retention	1 (0.2)	0 (0.0)	1 (0.1)
Reproductive system and breast disorders	3 (0.5)	0 (0.0)	3 (0.3)
Benign prostatic hyperplasia	2 (0.4)	0 (0.0)	2 (0.2)
Prostatomegaly	1 (0.2)	0 (0.0)	1 (0.1)

Data Source: Listing 16.2.3.1, Listing 16.2.7.1
A treatment-emergent sign or symptom (TESS) is defined as an adverse event that either (1) begins on or after the date of the first dose of study drug of E2020-G000-328 [up to 30 days after date of last dose of study drug of E2020-G000-328] or (2) increases in severity during the treatment period.
Subjects are counted only once per treatment in each row.
Number of subjects in Safety Population is used as the denominator for computing percentages.

Table 14.3.2.2.2
Summary of Serious Treatment-Emergent Signs or Symptoms by Body System and Preferred Term
Safety Population

	Lead-in Treatment Group		
Body System<br>Preferred Term	Donepezil SR 23 mg<br>n (%)	Donepezil IR 10 mg<br>n (%)	Total<br>n (%)
Number of Subjects	570	332	902
Number of subjects with serious treatment-emergent signs or symptoms	80 (14.0)	56 (16.9)	136 (15.1)
Respiratory, thoracic and mediastinal disorders	7 (1.2)	3 (0.9)	10 (1.1)
Choking	1 (0.2)	0 (0.0)	1 (0.1)
Chronic obstructive pulmonary disease	1 (0.2)	1 (0.3)	2 (0.2)
Dyspnoea	3 (0.5)	0 (0.0)	3 (0.3)
Lung disorder	1 (0.2)	0 (0.0)	1 (0.1)
Pleural effusion	1 (0.2)	0 (0.0)	1 (0.1)
Pulmonary embolism	0 (0.0)	2 (0.6)	2 (0.2)
Vascular disorders	2 (0.4)	1 (0.3)	3 (0.3)
Deep vein thrombosis	1 (0.2)	0 (0.0)	1 (0.1)
Hypertension	0 (0.0)	1 (0.3)	1 (0.1)
Hypotension	1 (0.2)	0 (0.0)	1 (0.1)

Data Source: Listing 16.2.3.1, Listing 16.2.7.1
A treatment-emergent sign or symptom (TESS) is defined as an adverse event that either (1) begins on or after the date of the first dose of study drug of E2020-G000-328 [up to 30 days after date of last dose of study drug of E2020-G000-328] or (2) increases in severity during the treatment period.
Subjects are counted only once per treatment in each row.
Number of subjects in Safety Population is used as the denominator for computing percentages.

tad_ae.sas 25MAY2010 22:15
