# Supplementary material for: Long-term safety and tolerability of donepezil 23 mg in patients with moderate to severe Alzheimer’s disease
Source: BMC Res Notes. 2012 Jun 8;5:283. doi: 10.1186/1756-0500-5-283 (PMC3493328; doi:10.1186/1756-0500-5-283)
Supplement: Additional file 4 — Subject Baseline Demographic Characteristics (Safety Population). [file 1756-0500-5-283-S4.pdf]

Table 14.1.2.1.2
Subject Baseline Demographic Characteristics
Safety Population

	Lead-in Treatment Group								
	Donepezil SR 23 mg			Donepezil IR 10 mg			Total		
	Male	Female	Total	Male	Female	Total	Male	Female	Total
Number of Subjects	197	373	570	130	202	332	327	575	902
Age (years) [1]									
N	197	373	570	130	202	332	327	575	902
Mean (SD)	73.4 (8.00)	74.5 (8.99)	74.1 (8.67)	73.5 (8.19)	75.2 (8.56)	74.5 (8.44)	73.4 (8.06)	74.7 (8.84)	74.3 (8.58)
Median	74.0	76.0	75.0	74.0	77.0	76.0	74.0	76.0	76.0
Min, Max	54, 90	47, 90	47, 90	52, 90	52, 91	52, 91	52, 90	47, 91	47, 91
Age in Years [1]: n (%)									
45 - 64	32 (16.2)	60 (16.1)	92 (16.1)	20 (15.4)	30 (14.9)	50 (15.1)	52 (15.9)	90 (15.7)	142 (15.7)
65 - 74	68 (34.5)	103 (27.6)	171 (30.0)	48 (36.9)	48 (23.8)	96 (28.9)	116 (35.5)	151 (26.3)	267 (29.6)
75 - 84	86 (43.7)	168 (45.0)	254 (44.6)	52 (40.0)	102 (50.5)	154 (46.4)	138 (42.2)	270 (47.0)	408 (45.2)
>=85	11 (5.6)	42 (11.3)	53 (9.3)	10 (7.7)	22 (10.9)	32 (9.6)	21 (6.4)	64 (11.1)	85 (9.4)
Gender: n (%)									
Male	197 (100.0)	0 (0.0)	197 (34.6)	130 (100.0)	0 (0.0)	130 (39.2)	327 (100.0)	0 (0.0)	327 (36.3)
Female	0 (0.0)	373 (100.0)	373 (65.4)	0 (0.0)	202 (100.0)	202 (60.8)	0 (0.0)	575 (100.0)	575 (63.7)

Data Source: Listing 16.2.4.1.2
[1] Age at baseline visit of Study E2020-G000-328.
SD = standard deviation.

tad_demo2.sas 25MAY2010 22:16

Table 14.1.2.1.2
Subject Baseline Demographic Characteristics
Safety Population

	Lead-in Treatment Group						Total		
	Donepezil SR 23 mg			Donepezil IR 10 mg					
	Male	Female	Total	Male	Female	Total	Male	Female	Total
Number of Subjects	197	373	570	130	202	332	327	575	902
Race: n (%)									
Black	5 (2.5)	11 (2.9)	16 (2.8)	2 (1.5)	5 (2.5)	7 (2.1)	7 (2.1)	16 (2.8)	23 (2.5)
White	151 (76.6)	278 (74.5)	429 (75.3)	92 (70.8)	153 (75.7)	245 (73.8)	243 (74.3)	431 (75.0)	674 (74.7)
Hispanic	7 (3.6)	35 (9.4)	42 (7.4)	6 (4.6)	12 (5.9)	18 (5.4)	13 (4.0)	47 (8.2)	60 (6.7)
Native American	0 (0.0)	0 (0.0)	0 (0.0)	0 (0.0)	0 (0.0)	0 (0.0)	0 (0.0)	0 (0.0)	0 (0.0)
Asian/Pacific	33 (16.8)	47 (12.6)	80 (14.0)	30 (23.1)	30 (14.9)	60 (18.1)	63 (19.3)	77 (13.4)	140 (15.5)
Other	1 (0.5)	2 (0.5)	3 (0.5)	0 (0.0)	2 (1.0)	2 (0.6)	1 (0.3)	4 (0.7)	5 (0.6)
Weight (kg): n (%)									
<55	12 (6.1)	119 (31.9)	131 (23.0)	10 (7.7)	66 (32.7)	76 (22.9)	22 (6.7)	185 (32.2)	207 (22.9)
55 to <65	38 (19.3)	107 (28.7)	145 (25.4)	23 (17.7)	63 (31.2)	86 (25.9)	61 (18.7)	170 (29.6)	231 (25.6)
65 to <75	53 (26.9)	96 (25.7)	149 (26.1)	36 (27.7)	45 (22.3)	81 (24.4)	89 (27.2)	141 (24.5)	230 (25.5)
>=75	94 (47.7)	51 (13.7)	145 (25.4)	61 (46.9)	28 (13.9)	89 (26.8)	155 (47.4)	79 (13.7)	234 (25.9)

Data Source: Listing 16.2.4.1.2
[1] Age at baseline visit of Study E2020-G000-328.
SD = standard deviation.

tad_demo2.sas 25MAY2010 22:16

Table 14.1.2.1.2
Subject Baseline Demographic Characteristics
Safety Population

	Lead-in Treatment Group								
	Donepezil SR 23 mg			Donepezil IR 10 mg			Total		
	Male	Female	Total	Male	Female	Total	Male	Female	Total
Number of Subjects	197	373	570	130	202	332	327	575	902
Body Mass Index									
N	197	371	568	129	201	330	326	572	898
Mean (SD)	25.6 (4.04)	24.8 (4.38)	25.1 (4.28)	25.5 (4.02)	25.0 (4.45)	25.2 (4.29)	25.6 (4.03)	24.9 (4.41)	25.1 (4.28)
Median	25.6	24.1	24.6	25.4	24.7	24.9	25.5	24.4	24.7
Min, Max	14, 48	14, 42	14, 48	19, 38	15, 42	15, 42	14, 48	14, 42	14, 48
Education (years)									
N	195	368	563	130	201	331	325	569	894
Mean (SD)	12.6 (4.07)	10.1 (4.29)	11.0 (4.38)	11.9 (4.29)	10.2 (4.31)	10.9 (4.38)	12.3 (4.17)	10.1 (4.29)	10.9 (4.38)
Median	12.0	11.0	12.0	12.0	12.0	12.0	12.0	11.0	12.0
Min, Max	0, 25	0, 20	0, 25	0, 22	0, 20	0, 22	0, 25	0, 20	0, 25

Data Source: Listing 16.2.4.1.2
[1] Age at baseline visit of Study E2020-G000-328.
SD = standard deviation.

tad_demo2.sas 25MAY2010 22:16

Table 14.1.2.1.2
Subject Baseline Demographic Characteristics
Safety Population

	Lead-in Treatment Group								
	Donepezil SR 23 mg			Donepezil IR 10 mg			Total		
	Male	Female	Total	Male	Female	Total	Male	Female	Total
Number of Subjects	197	373	570	130	202	332	327	575	902
Education in Years: n (%)									
0 - 8	33 (16.8)	127 (34.0)	160 (28.1)	30 (23.1)	63 (31.2)	93 (28.0)	63 (19.3)	190 (33.0)	253 (28.0)
9 - 11	38 (19.3)	68 (18.2)	106 (18.6)	20 (15.4)	31 (15.3)	51 (15.4)	58 (17.7)	99 (17.2)	157 (17.4)
12	36 (18.3)	94 (25.2)	130 (22.8)	30 (23.1)	63 (31.2)	93 (28.0)	66 (20.2)	157 (27.3)	223 (24.7)
13 - 16	66 (33.5)	63 (16.9)	129 (22.6)	33 (25.4)	37 (18.3)	70 (21.1)	99 (30.3)	100 (17.4)	199 (22.1)
> 16	22 (11.2)	16 (4.3)	38 (6.7)	17 (13.1)	7 (3.5)	24 (7.2)	39 (11.9)	23 (4.0)	62 (6.9)
Missing	2 (1.0)	5 (1.3)	7 (1.2)	0 (0.0)	1 (0.5)	1 (0.3)	2 (0.6)	6 (1.0)	8 (0.9)

Data Source: Listing 16.2.4.1.2
[1] Age at baseline visit of Study E2020-G000-328.
SD = standard deviation.

tad_demo2.sas 25MAY2010 22:16

Table 14.1.2.1.2
Subject Baseline Demographic Characteristics
Safety Population

	Lead-in Treatment Group								
	Donepezil SR 23 mg			Donepezil IR 10 mg			Total		
	Male	Female	Total	Male	Female	Total	Male	Female	Total
Number of Subjects	197	373	570	130	202	332	327	575	902
Type of Residence: n (%)									
Lives alone	1 (0.5)	15 (4.0)	16 (2.8)	3 (2.3)	14 (6.9)	17 (5.1)	4 (1.2)	29 (5.0)	33 (3.7)
Lives with caregiver	183 (92.9)	282 (75.6)	465 (81.6)	106 (81.5)	152 (75.2)	258 (77.7)	289 (88.4)	434 (75.5)	723 (80.2)
Lives with relative, or friend	8 (4.1)	46 (12.3)	54 (9.5)	14 (10.8)	17 (8.4)	31 (9.3)	22 (6.7)	63 (11.0)	85 (9.4)
Resides in adult/senior residence or retirement home	3 (1.5)	4 (1.1)	7 (1.2)	0 (0.0)	3 (1.5)	3 (0.9)	3 (0.9)	7 (1.2)	10 (1.1)
Resides in assisted living (independent/no skilled nursing)	1 (0.5)	14 (3.8)	15 (2.6)	4 (3.1)	12 (5.9)	16 (4.8)	5 (1.5)	26 (4.5)	31 (3.4)
Resides in intermediate nursing care facility	0 (0.0)	1 (0.3)	1 (0.2)	1 (0.8)	0 (0.0)	1 (0.3)	1 (0.3)	1 (0.2)	2 (0.2)
Resides in skilled nursing facility	0 (0.0)	8 (2.1)	8 (1.4)	1 (0.8)	2 (1.0)	3 (0.9)	1 (0.3)	10 (1.7)	11 (1.2)
Other	1 (0.5)	3 (0.8)	4 (0.7)	1 (0.8)	2 (1.0)	3 (0.9)	2 (0.6)	5 (0.9)	7 (0.8)

Data Source: Listing 16.2.4.1.2
[1] Age at baseline visit of Study E2020-G000-328.
SD = standard deviation.

tad_demo2.sas 25MAY2010 22:16
