# Supplementary material for: Long-term safety and tolerability of donepezil 23 mg in patients with moderate to severe Alzheimer’s disease
Source: BMC Res Notes. 2012 Jun 8;5:283. doi: 10.1186/1756-0500-5-283 (PMC3493328; doi:10.1186/1756-0500-5-283)
Supplement: Additional file 5 — Summary of Treatment Duration (Safety Population). [file 1756-0500-5-283-S5.pdf]

Table 14.3.8
Summary of Treatment Duration
Safety Population

	Lead-in Treatment Group					
	Donepezil SR 23 mg		Donepezil IR 10 mg		Total	
	From the First Dose Date of 326	From the First Dose Date of 328	From the First Dose Date of 326	From the First Dose Date of 328	From the First Dose Date of 326	From the First Dose Date of 328
Number of subjects	570	570	332	332	902	902
Treatment Duration (Months)						
n	569	569	332	332	901	901
Mean (SD)	16.21 (3.18)	10.59 (3.17)	15.41 (3.81)	9.79 (3.82)	15.92 (3.44)	10.30 (3.45)
Median	17.67	12.07	17.57	12.00	17.63	12.03
Min, Max	5.9, 18.9	0.3, 13.3	5.5, 18.9	0.0, 13.4	5.5, 18.9	0.0, 13.4
Duration in Months: n (%)						
< 3	0 (0.0)	34 (6.0)	0 (0.0)	38 (11.4)	0 (0.0)	72 (8.0)
3 to <6	1 (0.2)	32 (5.6)	7 (2.1)	23 (6.9)	8 (0.9)	55 (6.1)
6 to <9	44 (7.7)	33 (5.8)	35 (10.5)	29 (8.7)	79 (8.8)	62 (6.9)
9 to <12	37 (6.5)	146 (25.6)	28 (8.4)	73 (22.0)	65 (7.2)	219 (24.3)
12 to <15	32 (5.6)	324 (56.8)	26 (7.8)	169 (50.9)	58 (6.4)	493 (54.7)
15 to <18	408 (71.6)	0 (0.0)	216 (65.1)	0 (0.0)	624 (69.2)	0 (0.0)
>=18	47 (8.2)	0 (0.0)	20 (6.0)	0 (0.0)	67 (7.4)	0 (0.0)
Missing	1 (0.2)	1 (0.2)	0 (0.0)	0 (0.0)	1 (0.1)	1 (0.1)

Data Source: Listing 16.2.5.1
The treatment duration is defined as the total number of dosing days from first to last day (inclusive) of study treatment.
The duration in months is calculated as the treatment duration in days / 30 days.
Number of subjects in the Safety Population is used as the denominator for computing percentages.
SD = standard deviation.

tad_td.sas 25MAY2010 22:19

Ad hoc Table 14.3.8
Summary of Treatment Duration
Safety Population
Excluding Site 7139

	Lead-in Treatment Group					
	Donepezil SR 23 mg		Donepezil IR 10 mg		Total	
	From the First Dose Date of 326	From the First Dose Date of 328	From the First Dose Date of 326	From the First Dose Date of 328	From the First Dose Date of 326	From the First Dose Date of 328
Number of subjects	569	569	330	330	899	899
Treatment Duration (Months)						
n	569	569	330	330	899	899
Mean (SD)	16.21 (3.18)	10.59 (3.17)	15.47 (3.75)	9.85 (3.76)	15.94 (3.42)	10.32 (3.42)
Median	17.67	12.07	17.57	12.00	17.63	12.03
Min, Max	5.9, 18.9	0.3, 13.3	5.5, 18.9	0.0, 13.4	5.5, 18.9	0.0, 13.4
Duration in Months: n (%)						
< 3	0 (0.0)	34 (6.0)	0 (0.0)	36 (10.9)	0 (0.0)	70 (7.8)
3 to <6	1 (0.2)	32 (5.6)	5 (1.5)	23 (7.0)	6 (0.7)	55 (6.1)
6 to <9	44 (7.7)	33 (5.8)	35 (10.6)	29 (8.8)	79 (8.8)	62 (6.9)
9 to <12	37 (6.5)	146 (25.7)	28 (8.5)	73 (22.1)	65 (7.2)	219 (24.4)
12 to <15	32 (5.6)	324 (56.9)	26 (7.9)	169 (51.2)	58 (6.5)	493 (54.8)
15 to <18	408 (71.7)	0 (0.0)	216 (65.5)	0 (0.0)	624 (69.4)	0 (0.0)
>=18	47 (8.3)	0 (0.0)	20 (6.1)	0 (0.0)	67 (7.5)	0 (0.0)

Data Source: Listing 16.2.5.1
The treatment duration is defined as the total number of dosing days from first to last day (inclusive) of study treatment.
The duration in months is calculated as the treatment duration in days / 30 days.
Number of subjects in the Safety Population is used as the denominator for computing percentages.
SD = standard deviation.

tad_td.sas 15JUN2010 23:01
